# *Mettl3*-Mediated m^6^A Modification is Essential for Visual Function and Retinal Photoreceptor Survival

**DOI:** 10.1167/iovs.65.14.40

**Published:** 2024-12-27

**Authors:** Xiaoyan Jiang, Kuanxiang Sun, Yudi Fan, Qianchun Xiang, Rong Zou, Yeming Yang, Xianjun Zhu, Wenjing Liu

**Affiliations:** 1 The Sichuan Provincial Key Laboratory for Human Disease Gene Study and Center for Medical Genetics, Sichuan Provincial People′s Hospital, University of Electronic Science and Technology of China, Chengdu, China; 2 Henan Branch of National Clinical Research Center for Ocular Diseases, Henan Eye Hospital, People′s Hospital of Zhengzhou University, Henan Provincial People′s Hospital, Zhengzhou, China; 3 Qinghai Key Laboratory of Qinghai Tibet Plateau Biological Resources, Chinese Academy of Sciences and Qinghai Provincial Key Laboratory of Tibetan Medicine Research, Northwest Institute of Plateau Biology, Xining, China; 4 Research Unit for Blindness Prevention of Chinese Academy of Medical Sciences (2019RU026), Sichuan Academy of Medical Sciences and Sichuan Provincial People′s Hospital, Chengdu, China

**Keywords:** N6-methyladenosine (m^6^A), METTL3, retinal photoreceptors, knockout mouse models, retinal degeneration

## Abstract

**Purpose:**

N6-methyladenosine (m^6^A) modification, one of the most common epigenetic modifications in eukaryotic mRNA, has been shown to play a role in the development and function of the mammalian nervous system by regulating the biological fate of mRNA. METTL3, the catalytically active component of the m^6^A methyltransferase complex, has been shown to be essential in development of in the retina. However, its role in the mature retina remains elusive. In this study we aim to investigate the in vivo function of *Mettl3* in the photoreceptor cells using a conditional knockout allele of *Mettl3*.

**Methods:**

Deletion of *Mettl3* in rod cells led to progressive retinal degeneration, including progressive retinal thinning, impaired visual function, shortened photoreceptor outer segments (OS), and reduced expression of disk membrane proteins. Similarly, *Mettl3* deficiency in cone cells led to the gradual degeneration of cone opsins. Additionally, *Mettl3* knockout significantly decreased the expression of the METTL14 subunit and overall m^6^A methylation levels in the retina.

**Results:**

Multi-omics analyses revealed that *Mettl3* deletion led to the downregulation of mRNA and protein levels of 10 key target genes in rod cells, ultimately resulting in the progressive death of photoreceptors. *Mettl3* controls expression of its target genes by regulating their m^6^A modification, ultimately leading to rod cell death.

**Conclusions:**

These findings highlight critical roles of METTL3 in maintaining retinal photoreceptor function and further elucidate the mechanisms of m^6^A modification in photoreceptors.

N6-methyladenosine (m^6^A) modification is one of the most common and widely studied modifications of eukaryotic messenger RNAs (mRNAs). Modification of m^6^A is a dynamic and reversible process mediated by a series of enzymes that can be categorized as “writers,” “erasers,” and “readers.” The “writers” are mainly complexes of m^6^A methyltransferases, including METTL3, METTL14, WTAP, VIRMA, and HAKAI, whose main function is to catalyze the methylation of adenosine residues.^1^^,^^2^ “Scavengers” are demethylating enzymes, such as FTO and ALKBH5, which are mainly responsible for removing methyl groups.^1^^,^^3^ “Readers” are m^6^A-binding proteins, such as YTHDF1, YTHDF2, YTHDC2 and IGF2BP2, which recognize and bind mRNAs undergoing m^6^A modification, thereby regulating mRNA egress, splicing, translation, stability, and degradation.^4^ Studies have shown that m^6^A modification plays a key role in many biological processes, such as embryonic development, stem cell differentiation, and circadian regulation.^5^^–^^8^ Aberrant m^6^A modifications have been associated with a variety of diseases, including cancer, neurological disorders, and metabolic disorders, highlighting the potential of m^6^A modifications as a therapeutic strategy for these diseases.^9^^–^^13^

METTL3, as the only subunit in the m^6^A methyltransferase complex with methyltransferase catalytic activity, has become a key regulator of mRNA methylation.^14^ Numerous studies have shown that METTL3 plays an important role in cellular processes such as cell cycle progression, apoptosis, and differentiation. For example, in various cancers, METTL3 has been shown to promote the translation of specific oncogenes, thereby promoting tumorigenesis and cancer progression.^15^^,^^16^ In leukemia, METTL3-mediated m^6^A modification enhances the stability and translation of MYC and BCL2 mRNAs, promoting leukemogenesis.^17^^,^^18^ Similarly, in glioblastoma, METTL3 promotes tumor growth and self-renewal of cancer stem cells through m^6^A modification of SOX2 mRNA.^19^^,^^20^ These findings highlight the critical role of METTL3 in cancer biology and suggest that targeting METTL3 may be a potential therapeutic approach. In addition to cancer, METTL3 has been linked to various other diseases, including obesity, diabetes, cardiovascular disease, and immune disorders.^21^^–^^23^

Photoreceptor cells in the retina include rod and cone cells, which are essential for converting light into neural signals that enable vision. Retinitis pigmentosa (RP) is a group of inherited retinal degenerative diseases characterized by a progressive loss of photoreceptor cells, leading to impaired vision and ultimately blindness.^24^^,^^25^ Over 100 genes have been identified as RP-causing genes, which are involved in a variety of cellular processes, including phototransduction, RNA splicing, and protein transport.^24^^–^^29^ Despite the diversity of genes, the common result is photoreceptor degeneration. Current treatments for photoreceptor degeneration (e.g., gene therapy and stem cell therapy) have made great strides and offer promising avenues for treating RP.^30^^–^^33^ However, effective treatments are still limited, so it is crucial to search for new causative genes and explore the pathogenic mechanisms.

Currently identified RP-causing genes explain only about 70% of RP patients, and the causative mechanism has not been clarified in the remaining 30% of RP patients.^25^^,^^34^ This is likely to be caused by nonclassical genetic variants (including variants in non-coding regions, complex variants and epigenetic variants). Modifications of m^6^A are gaining attention as key modifications in epigenetics. Recent studies have begun to recognize the role of m^6^A modifications in the retina, and it has been shown that METTL14 is essential for the normal function and survival of photoreceptor cells and that METTL14 deficiency affects the expression of genes essential for visual signal processing and for the development and maintenance of photoreceptor cilia.^35^ However, the function of METTL3 in photoreceptor cells has not been extensively studied. Given that METTL3 is involved in regulating processes such as mRNA stability and translation in other cell types, we speculate that METTL3 may similarly affect the fate of downstream target genes in photoreceptor cells.

The objective of this study is to dissect in vivo functions of METTL3 in photoreceptor cells. Our data revealed that deletion of *Mettl3* in rod and cone cells resulted in progressive retinal degeneration. *Mettl3* deficiency diminished the expression of METTL14 and m^6^A methylation levels in the retina. Moreover, *Mettl3* influences expression levels of its target genes by regulating their m^6^A modification, ultimately leading to neuronal cell death. These data highlight essential roles of METTL3 as a core subunit of the m^6^A methyltransferase complex in maintaining retinal photoreceptor function.

## Material and Methods

### Mouse Models and Genotyping

*Mettl3^flox/flox^* mice in C57BL/6J background were purchased from Shanghai Model Organisms. Rod-Cre mice were obtained from The Jackson Laboratory, Bar Harbor, ME, USA (B6.Cg-Pde6b^+^Tg(Rho-icre)1Ck/Boc, https://www.jax.org/strain/015850).^36^ Cone-Cre mice were kindly provided by Professor Yun-zheng Le from the University of Oklahoma Health Sciences Center, Oklahoma City, OK, USA.^37^ Rod-specific *Mettl3* knockout (RKO) and cone-specific *Mettl3* knockout (CKO) were generated by breeding *Mettl3^flox/flox^* mice with Rod-Cre or Cone-Cre mice. To monitor Cone-cre expression, we crossed ROSA26-tdTomato reporter to CKO mice.^38^ ROSA26-tdTomato reporter is a Cre reporter tool strain containing a loxP-flanked STOP cassette preventing transcription of a CAG promoter-driven red fluorescent protein variant (tdTomato, https://www.jax.org/strain/007914). The reporter was inserted into the Gt(ROSA)26Sor locus. ROSA26-tdTomato reporter mice express strong tdTomato fluorescence after Cre-mediated recombination.

All experimental protocols involving animals were approved by the Institutional Review Board of Sichuan Provincial People′s Hospital and were conducted in accordance with the ARVO Statement for the Use of Animals in Ophthalmic and Vision Research. Animals were kept in a specific pathogen-free (SPF) environment with temperature and relative humidity maintained at approximately 25°C and 50%, respectively, under a 12-hour light/12-hour dark cycle.

Genotyping was performed using extracted mouse tail DNA by PCR amplification. Primer sequences used for mouse genotyping are as follows. *Mettl3*^flox^: *Mettl3*-loxp-F, 5′-CCCAACAGAGAAACGGTGAG-3′; *Mettl3*-loxp-R, 5′-GGGTTCAACTGTCCAGCATC-3′. Rho-Cre: RH1, 5′-TCAGTGCCTGGAGTTGCGCTGTGG-3′; iCre, 5′-CTTAAAGGCCAGGGCCTGCTTGGC-3′. Cone-Cre: Cre-F, 5′-GAACGCACTGATTTCGACCA-3′; Cre-R: 5′-GCTAACCAGCGTTTTCGTTC-3′.

### Real-Time Quantitative Polymerase Chain Reaction (RT-qPCR)

RNA was extracted from mouse retinal tissue and reverse transcription to cDNA was performed using xx kit. The primer sequences used for RT-qPCR in this study are listed in Supplementary Table S1. After the reaction system was prepared, RT-qPCR was conducted with the following program: 95°C (30 s), [95°C (5 s), 60°C (30 s)] × 42 cycles, 12°C (Hold). *Gapdh* was used as an internal reference, and the relative expression levels of the corresponding genes were calculated using 2^−Δ(ΔCt)^ formula.

### Western Blot

Proteins were extracted from mouse retinal tissue and protein concentration was determined spectrophotometrically with a BCA assay kit. SDS-PAGE electrophoresis was performed using FuturePAGE precast gels (Ace Biotechnology, Hunan, China). It was then transferred to the NC membrane at 100 V, 280 mA for a duration depending on the molecular weight of the target protein (60–120 minutes). The membrane is sealed with a sealing solution (8% skimmed milk or 5% BSA solution) on a shaker for one hour at room temperature. Primary antibody diluted with the sealing solution was added and incubated overnight at 4°C on a shaker. The membrane was washed four times with 1 × Tris-buffered saline solution with Tween 20 (TBST) at room temperature, incubated with secondary antibody diluted in the blocking solution for two hours at room temperature on a shaker, and washed four times with 1 × TBST at room temperature. Finally, the membrane was incubated with chemiluminescent substrate, and the target protein bands were visualized using a protein blotting imaging system. If additional primary antibody incubation is required, the membrane is incubated with an alkaline membrane wash buffer for 15 minutes at room temperature and then reconstituted with a sealing solution.

### Mouse Retinal Cryosectioning and Immunohistochemistry

Eye balls marked on the superior nasal position were enucleated and fixed overnight in 4% paraformaldehyde. The cornea was cut under a dissecting microscope and continued to be fixed on ice for two hours. The eyeballs were then dehydrated in 30% sucrose until they sank to the bottom. Under a dissecting microscope, the cornea and lens were removed, and the eyeball was placed in an embedding mold with OCT and frozen in a −80°C freezer for two hours. Cryosections were made at a thickness of 12 µm starting from the vicinity of the optic nerve, dried in an oven at 37°C for 30 minutes, circled with an immunohistochemistry pen on the retinal tissue sections, and washed with PBS. The sections were incubated with immunofluorescence blocking solution for two hours at room temperature, and the primary antibody diluted with immunofluorescence blocking solution was added and incubated overnight at 4°C. The next day, the sections were washed with PBS, with secondary antibody and DAPI added, and incubated for two hours at room temperature with humidification. The sections were removed, excess water was dried, sealing oil was added to the retinal tissue, and a coverslip was slowly placed over the section to avoid the formation of air bubbles. Images were captured using an LSM900 laser confocal microscope (Zeiss, Oberkochen, Germany) and saved for analysis. Primary and secondary antibodies used in experiment of Western blot and immunohistochemistry are listed in Supplementary Table S2.

### Retinal Flat Mount

Eyeballs were marked on the superior nasal side, enucleated and immersed in ice-cold 4% PFA for three hours. They were then immersed in PBS on ice for three hours. Subsequently, 0.4% PFA was added for further fixation for 24 hours. Under a dissecting microscope, the retina was isolated, divided into four quadrants, and flattened. The retina was incubated with ice-cold methanol for three minutes and then incubated with immunofluorescence blocking solution for two hours at room temperature. Primary antibody was added and incubated overnight at 4°C. The following day, the retina was washed with PBS, and secondary antibodies were added and incubated for four hours at room temperature. Under a dissecting microscope, the retina was laid flat on a slide and covered with mounting medium, and then a coverslip was slowly placed over the section. Images were captured using a Zeiss 900 laser confocal microscopy and saved for analysis.

### Mouse Retinal Paraffin Sectioning and Hematoxylin and Esosin (H&E) Staining

Labeled eye balls were removed, and immersed in eye fixative for two hours, followed by three rinses with PBS. Gradient dehydration was performed using a series of ethanol gradients ranging from low (70%) to high (100%) concentrations and cleared in xylene for 30 minutes at room temperature. The retinal tissue was then immersed in melted paraffin for three hours before being placed in an embedding mold containing melted paraffin and cooled in a refrigerator to solidify. Sections were cut using a paraffin microtome, then the slides were stained with H&E. Images were captured using a microscope and saved for analysis.

### Mouse Retinal Electrophysiology (ERG)

Animals were weighed to calculate the amount of anesthetic required (15 µL/g) and then placed in the ERG room for overnight dark acclimation. The next day, an anesthetic solution (1 mL ketamine + 50 µL xylazine hydrochloride) was prepared. The mice were anesthetized by intraperitoneal injection, and then a compound tropicamide pupil dilator was applied to the eyes. Dark adaptation tests were performed under different light intensities. After a period of light adaptation, photopic tests were carried out under different light intensities. At the end of the recording, antibiotic eye ointment was applied to both eyes of the mice, and then the mice were placed at 37°C for recovery.

### Dot Blot Assay

Total retinal RNA was extracted from the experimental group of mice, and mRNA was isolated from the total RNA using an mRNA purification kit. MRNA concentration was determined and adjusted to equalize the mRNA concentration in the experimental and control groups. MRNA 2 µL was spotted onto the NC membrane and crosslinked twice with 12,000 µJ of crosslinker. The membrane was then incubated with 5 mL of 5% BSA blocking solution for one hour at room temperature. The m^6^A antibody diluted in the blocking solution was then added and incubated overnight at 4 °C on a shaker. The secondary antibody diluted with the blocking solution was added and incubated on a shaker at room temperature for two hours. Spot images were acquired using a protein blotting imaging system. Methylene blue staining was performed on the NC membrane as a loading control.

### Proteomic Analysis

Proteomic analysis was conducted on the retinas of 2.5-month-old Ctrl and RKO mice with three independent biological replicates. Retinas from each animal were collected and immediately flash-frozen in liquid nitrogen. Samples were sent to Jingjie Bio-Tech Co., Ltd. (Hangzhou, Zhejiang Province, China) for protein extraction and LC-MS/MS analysis. Differential protein expression was determined using fold change thresholds greater than 1.2 as significant upregulation and less than 1/1.2 as significant downregulation, with a *P* value < 0.05. Data analysis was performed using bioinformatics tools.

### Statistical Analysis

Data analysis in this study was performed using GraphPad Prism 9.0 software, and statistical graphs were generated accordingly. Statistical analyses were conducted using t-tests for comparisons between two groups, with results presented as mean ± SEM. For comparisons among multiple groups, one-Way ANOVA was employed. A *P* value < 0.05 was considered statistically significant. Significance levels were denoted as follows: *P < 0.05, ** *P* < 0.01, ***P < 0.001, and ****P < 0.0001. Nonsignificant differences (*P* > 0.05) were indicated as “ns” (no significance).

## Results

### Generation of *Mettl3* Conditional Knockout Mice

**Figure 1.**
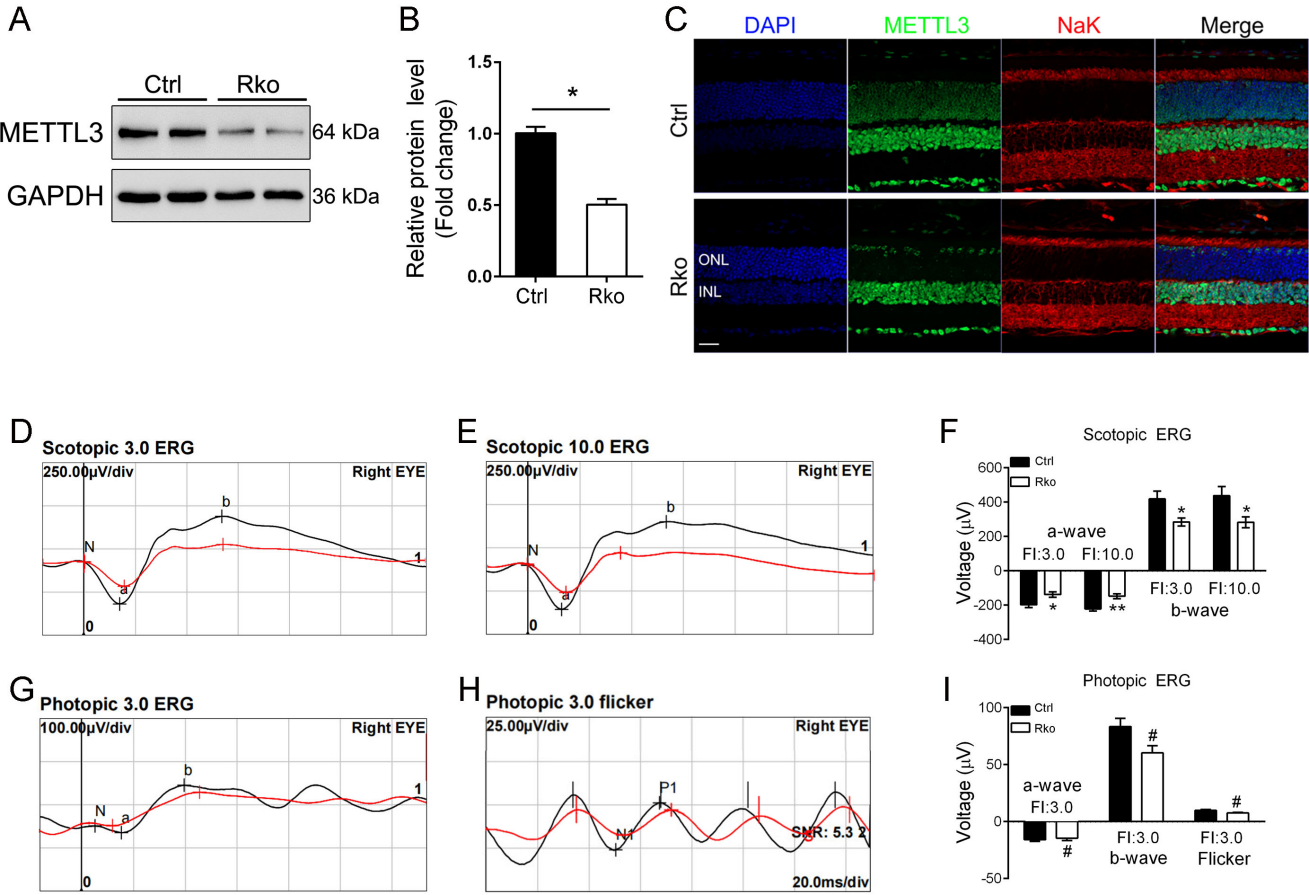
Specific deletion of *Mettl3* in retinal rods causes impaired visual function. (**A**, **B**) Western blot analysis of *Mettl3Rko retinas* of three-month-old Ctrl and RKO mice using an anti-METTL3 antibody revealed diminished expression level of METTL3. GAPDH served as the loading control. Grayscale values were used to quantify protein expression levels. (**C**) Immunofluorescence staining of frozen retinal sections from Ctrl and RKO mice revealed loss of METTL3 in rod cells. *Green* represents METTL3, marking their expression locations in the retina. *Red* represents NaK ATPase antibody labeled inner segments. *Scale bars:* 30 µm. (**D**, **E**) Waveforms recorded following photic stimulation at 3.0 and 10.0 cd · s/m^2^ under dark-adapted conditions for Ctrl and RKO mice, respectively. (**F**) Statistical analysis of the a- and b-wave amplitudes from waveforms of Ctrl and RKO mice depicted in (**D**, **E**). (**G**) Waveforms elicited by a 3.0 cd · s/m^2^ light stimulus under light-adapted conditions. (**H**) Flicker light response under light-adapted conditions. (**I**) Statistical analysis of the a- and b-wave amplitudes, as well as the difference between the peak and trough of the flicker response, for Ctrl and RKO mice illustrated in (**G**, **H**). *P < 0.05, **P < 0.01, #*P* > 0.05.

METTL3 was highly expressed in the inner nuclear layer (INL) and outer nuclear layer (ONL) of the mouse retina (Supplementary Fig. S1A), suggesting that *Mettl3* may have potential functions in the retina. To investigate the function of *Mettl3* in rod cells, rod specific knockout model of *Mettl3* (*Mettl3^loxp/loxp^* Rho-Cre, named RKO) was generated by crossing Rho-Cre mice with *Mettl3^loxp/loxp^* mice (Supplementary Fig. S1B). Littermate *Mettl3^loxp/loxp^* mice were used as controls. Mice were genotyped using agarose gel electrophoresis (Supplementary Fig. S1C). The knockout efficiency of *Mettl3* in RKO retina was evaluated by protein immunoblotting, and results showed that the expression level of METTL3 in RKO mice was reduced by approximately 50% at three months of age (Figs. 1A, 1B). Immunostaining analysis revealed loss of METTL3 expression in retinal section of RKO mice at three months of age (Fig. 1C). Considering that rod cells account for about 60% of the entire number of retinal cells, METTL3 level was effectively reduced in rod cells of RKO mice.

### *Mettl3* is Essential for Rod Cell Survival and Retinal Function

**Figure 2.**
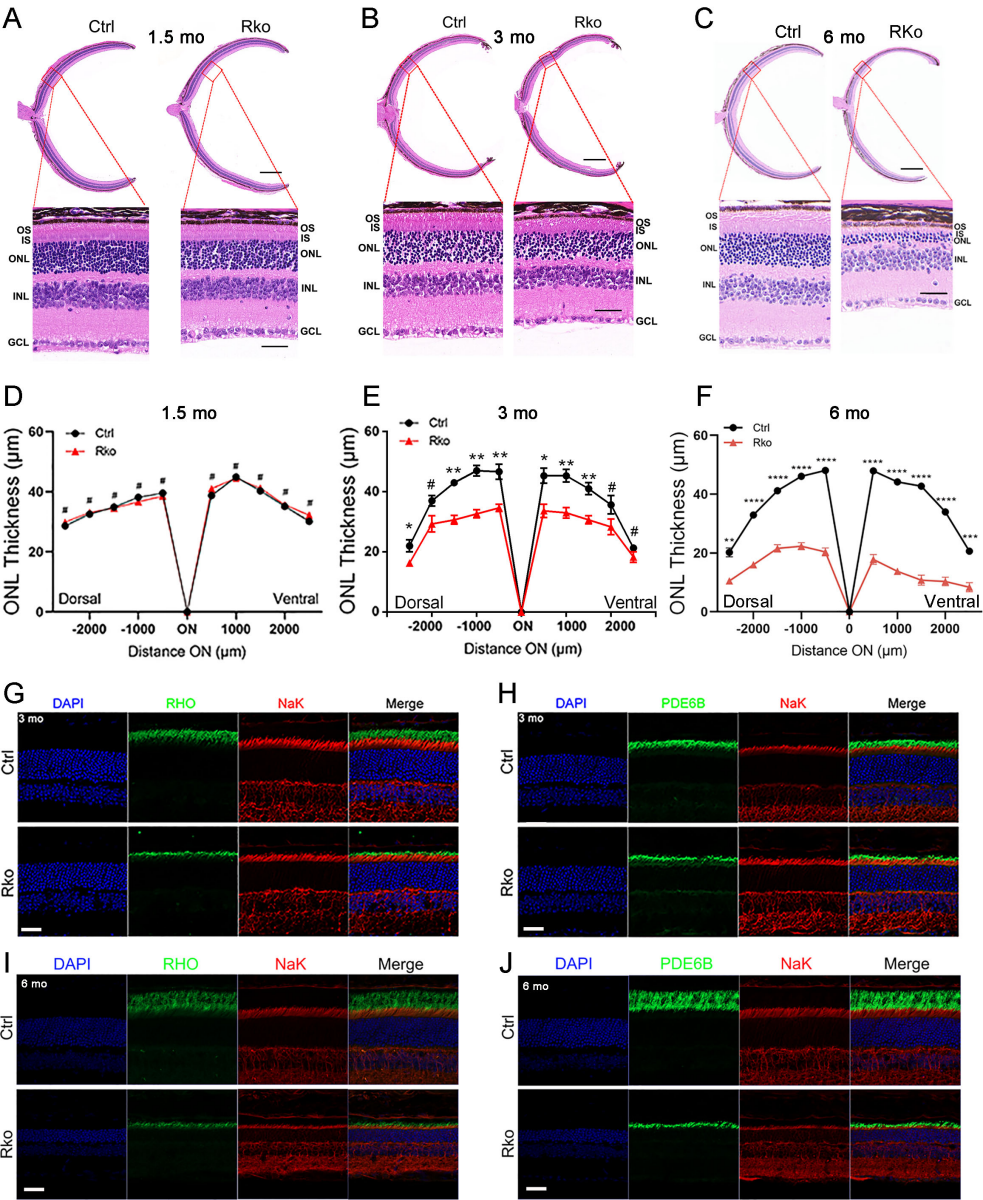
*Mettl3* deficiency in rod cells leads to retinal degeneration. (**A–C**) H&E staining of retinas from 1.5-, three-, six-month-old Ctrl and RKO mice. *Scale bar:* 50 µm. (**D–F**) Progressive reduction of the outer nuclear layer (ONL) thickness in RKO mice at three and six months of age. ONL was measured every 500 µm from the optic nerve head to both sides. Statistical significance: *n* = 3 per group. ****P < 0.001; ***P < 0.001; **P < 0.01; *P < 0.05; #*P* > 0.05. (**G**, **H**) Co-immunofluorescence staining of frozen retinal sections from three-month-old Ctrl and RKO mice. *Green* represents the disc proteins RHO (**G**) and PDE6B (**H**) labeling the outer segments and red indicates NaK ATPase labeling the inner segments. Nuclei were counterstained with DAPI. (**I**, **J**) Co-immunofluorescence staining of frozen retinal sections from six-month-old Ctrl and RKO mice. Green fluorescence labels the outer segment disc proteins RHO (**I**) and PDE6B (**J**), and red fluorescence marks NaK ATPase in the inner segments. Nuclei were counterstained with DAPI. *Scale bar:* 30 µm.

To assess whether visual function was altered in *Mettl3* RKO mice, we performed ERG examinations on three-month-old mice. Dark-adapted ERG examination revealed that the mean amplitudes of a-wave in RKO mice were decreased by ∼32% and ∼35% at light intensities of 3.0 cd · s/m^2^ and 10.0 cd · s/m^2^, respectively (Figs. 1D–F). The mean amplitudes of b-wave in RKO mice were decreased by ∼38% and ∼42% at light intensities of 3.0 cd · s/m^2^ and 10.0 cd · s/m^2^, respectively (Figs. 1D–F). Whereas light-adapted ERG examination did not reveal significant changes (Figs. 1G–I), indicating no obvious defect in the function of the cone cells in RKO mice at this time point. To further investigate the pathological changes in RKO mice, we performed H&E staining on paraffin sections of retinas from 1.5-, three-, six-month-old mice (Figs. 2A–C). Retinas of 1.5-month-old RKO mice did not show a degenerative phenotype (Fig. 2D). However, the thickness of the ONL of three-month-old RKO mice was reduced by approximately 25% (Fig. 2E). Moreover, the thickness of the ONL of six-month-old *Mettl3* RKO mice was further reduced by approximately 60% (Fig. 2F).

The phototransduction process is dependent on membrane disc proteins, which are crucial for retinal function. Abnormal expression or loss of function of these proteins can result in a range of retinal diseases, including RP.^39^^,^^40^ To investigate whether the protein expression of membrane disc proteins was affected by *Mettl3* deletion, we performed western blot assays on several membrane disc proteins, and results showed that the expression levels of several membrane disc proteins were significantly down-regulated at three months of age (Supplementary Figs. S2A–B), further revealing degenerative degradation of the OS of rod cells. Moreover, immunohistochemistry staining analysis with antibodies against major membrane disc proteins RHO and PDE6B revealed that the OS of rod cells in 3-month-old *Mettl3* RKO mice became shorter compared with that of control mice (Figs. 2G, 2H). At six months of age, thickness of OS and ONL of RKO mice was further reduced compared with that of controls (Figs. 2I, 2J). At eight months of age only trace number of cells were visible in ONL in RKO mice (Supplementary Fig. S3).

The retinal Müller glial cells are activated under pathological conditions and exhibits astrocyte proliferation.^41^^,^^42^ We examined GFAP, a marker protein for Müller glial cells, and found that GFAP expression was upregulated and exhibited Müller glial proliferation in RKO mice (Supplementary Figs. S4A–C). TUNEL staining assay revealed the presence of stain-positive cells in the ONL of RKO mice (Figs. S4D–E), suggesting that *Mettl3* deletion led to apoptosis of photoreceptor cells. These results indicated that *Mettl3* deletion led to diminished expression of membrane disc protein expression in rod cells, which triggers retinal degeneration and functional abnormalities. It is plausible that *Mettl3* ablation affected cell survival and function of rod cells and resulted in decreased expression of membrane disc proteins.

### CKO Mice Exhibit a Progressive Degenerative Phenotype of Cone Cells

**Figure 3.**
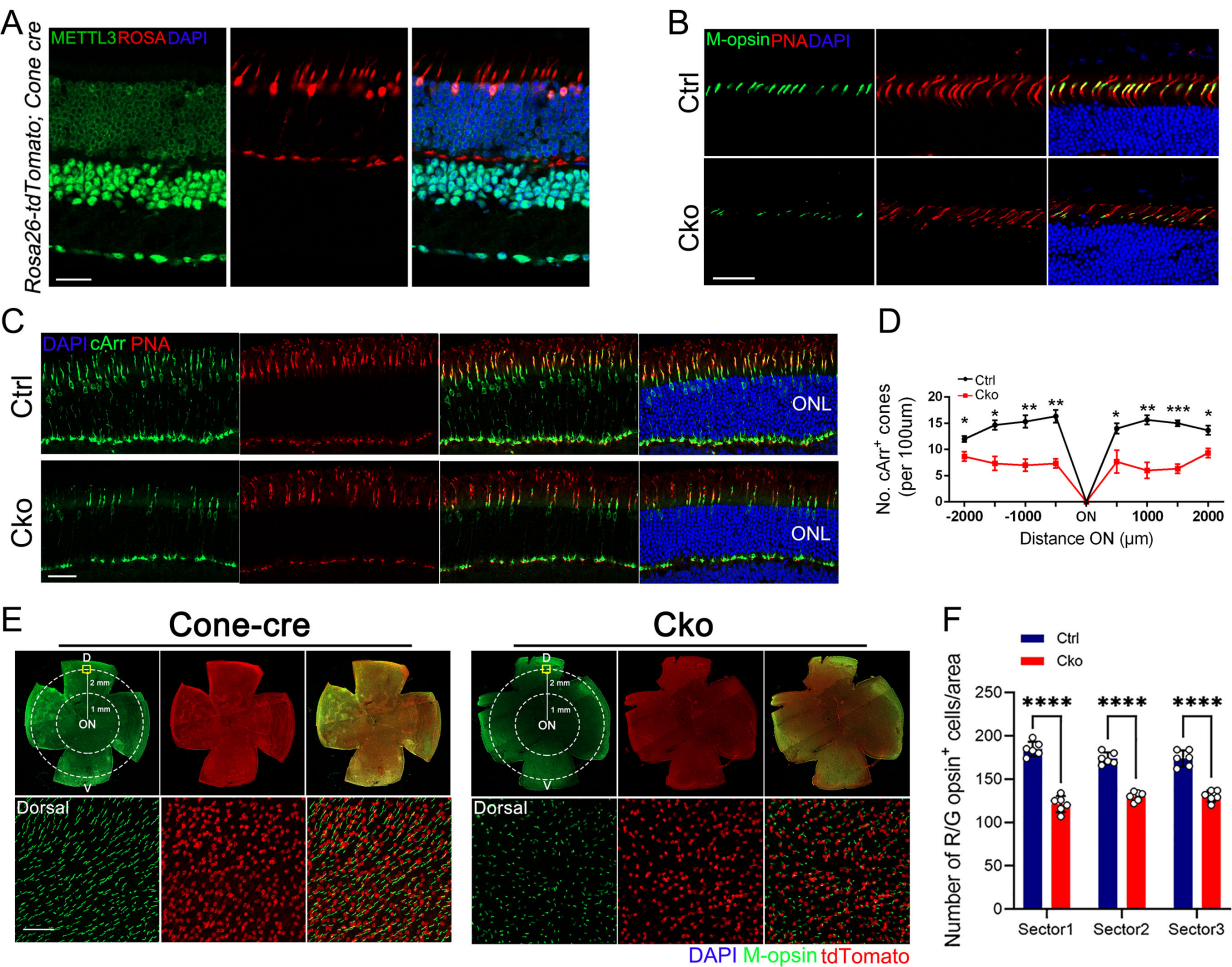
Cone photoreceptor-specific *Mettl3* knockout induces cone receptor degeneration at six months of age. (**A**) Immunofluorescence staining of retinal cryosections from Cone-Cre mice. *Green* indicates the expression of METTL3 protein in the retina, and *red* represents the fluorescence of ROSA-tdTomato, which represents Cre expression. Nuclei were counterstained with DAPI. *Scale bar:* 20 µm. (**B**) Retinal cryosections from six-month-old mice labeled with cone marker PNA and M-opsin. Nuclei counterstained with DAPI. *Scale bar:* 30 µm. (**C**) Representative immunofluorescence images of cone arrestin (*green*) and PNA (*red*) in the retina of six-month-old Ctrl and CKO mice. Nuclei counterstained with DAPI (*blue*). *Scale bar:* 20 µm. (**D**) In Ctrl and CKO retinas, the number of cone arrestin-labeled cones per 500 µm field of view. Cone clusters counted in the inferior and superior quadrants of the retina, starting −2000 µm from the ora serrata and moving toward the optic disc every 500 µm. (**E**) Immunostaining for M-opsin on retinal flat mounts from six-month-old Ctrl and CKO mice revealed a significant reduction in cone cell numbers in the CKO retina (**F**). with spontaneous red fluorescence from ROSA-tdTomato,. Scale bar, 50 µm. Representative images from the dorsal quadrant of the retina are shown below. Schematic of the retinal plane mount indicating the two sectors (radius: 1 and 2 mm) used for counting cones.

**Figure 4.**
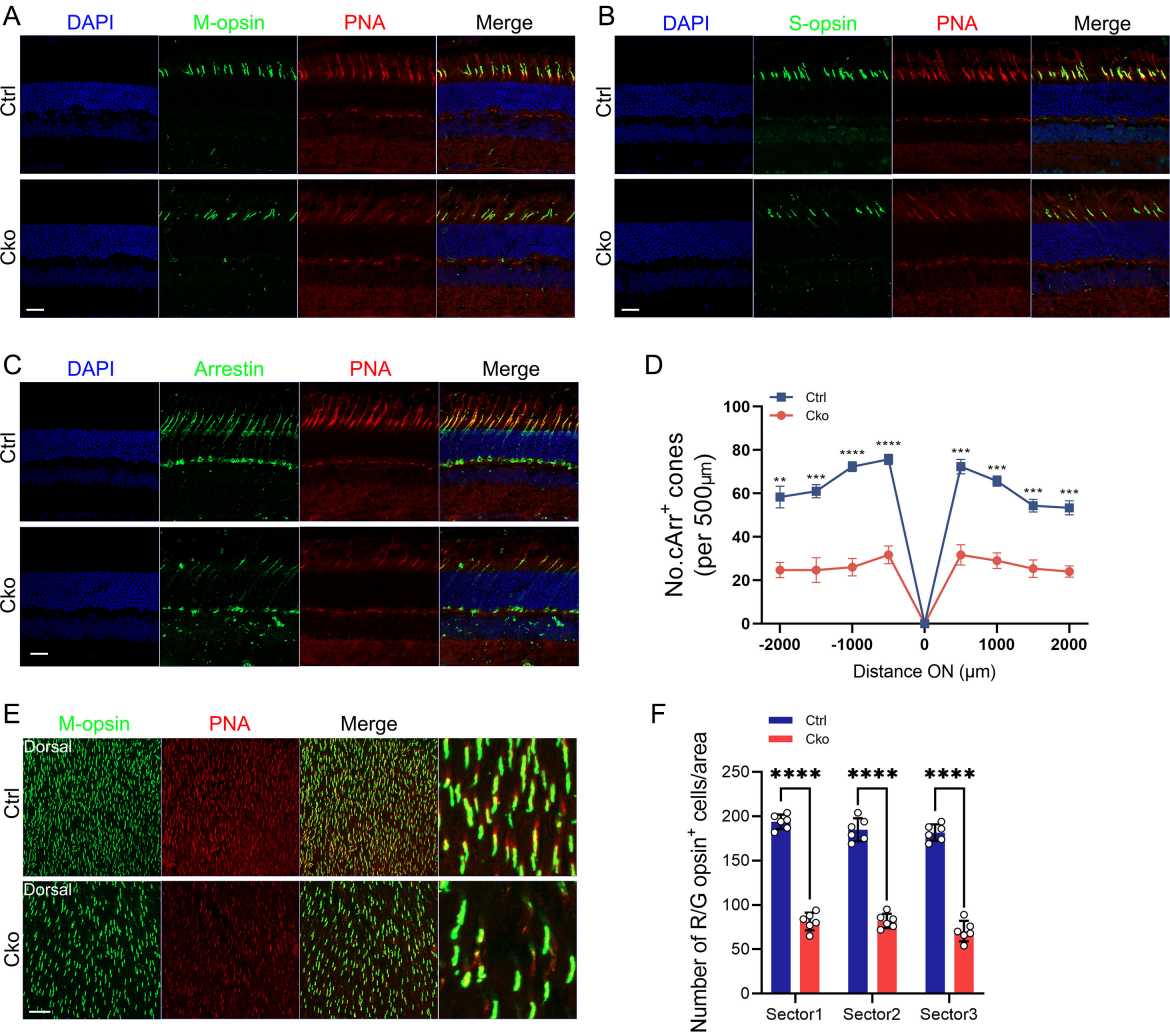
Severe degeneration of cone photoreceptors in 12-month-old CKO mice. (**A–C**) Immunofluorescence staining of retinal cryosections from 12-month-old mice using M-opsin (**A**), S-opsin (**B**), and Arrestin (**C**) to label cone photoreceptors, co-stained with the cone-specific marker PNA (*red*). Nuclei were counterstained with DAPI (*blue*). *Scale bar:* 30 µm. (**D**) Quantification of Arrestin-positive cone photoreceptors in both Ctrl and CKO retinas, counted across every 500 µm of retinal sections. **P < 0.01, ***P < 0.001, ****P < 0.0001. (**E**, **F**) Immunostaining of whole-mounted retinas from Ctrl and CKO mice using M-opsin (*green*) and PNA (*red*). Quantification shows a significant reduction in cone photoreceptor numbers in CKO retinas compared to controls. *Scale bar:* 30 µm. ****P < 0.0001.

Cone cells are primarily responsible for daytime vision and color vision.^43^^,^^44^ We generated CKO mice to explore the effects of *Mettl3* deficiency in cone cells. Here we introduced ROSA26-tdTomato reporter mice to monitor the specific expression of Cone-Cre in cone cells. ROSA26-tdTomato reporter mice express strong tdTomato fluorescence following Cone cre-mediated recombination (Fig. 3A). Immunostaining with L/M-opsin antibody and cone marker Peanut agglutinin (PNA) revealed evident loss of L/M-opsin-marked cone cells at six months of age (Fig. 3B). We then immunolabeled retinal cryosections with Cone-Arrestin (cArr) and Alexa Fluor-594-conjugated PNA. Immunofluorescence staining results showed a significant reduction in the number of cones in CKO mice (Figs. 3A–D). In addition, immunostaining for M-opsin on retinal flat mounts from six-month-old CKO mice revealed a significant reduction in cone cell numbers (Figs. 3E, 3F). Moreover, we also analysis cone degeneration in CKO mice at three, 3.5 and 12 months of age (Fig. 4). At 3 months of age, no visible cone degeneration was observed (Supplementary Fig. S5). Small reduction of Arrestin or M-opsin labeled cone cells was observed in CKO mice at 3.5 months of age (Supplementary Fig. S6). Moreover, a drastic decrease in the number of cone cells was observed in CKO retinas (Figs. 4C, 4D), which was consistent with the immunolabeling results of retinal flat mounts (Figs. 4E, 4F). The above results suggested that *Mettl3* is also essential for the survival of cone cells.

### Downregulation of Retinal m^6^A Modification Levels in *Mettl3* RKO Mice

**Figure 5.**
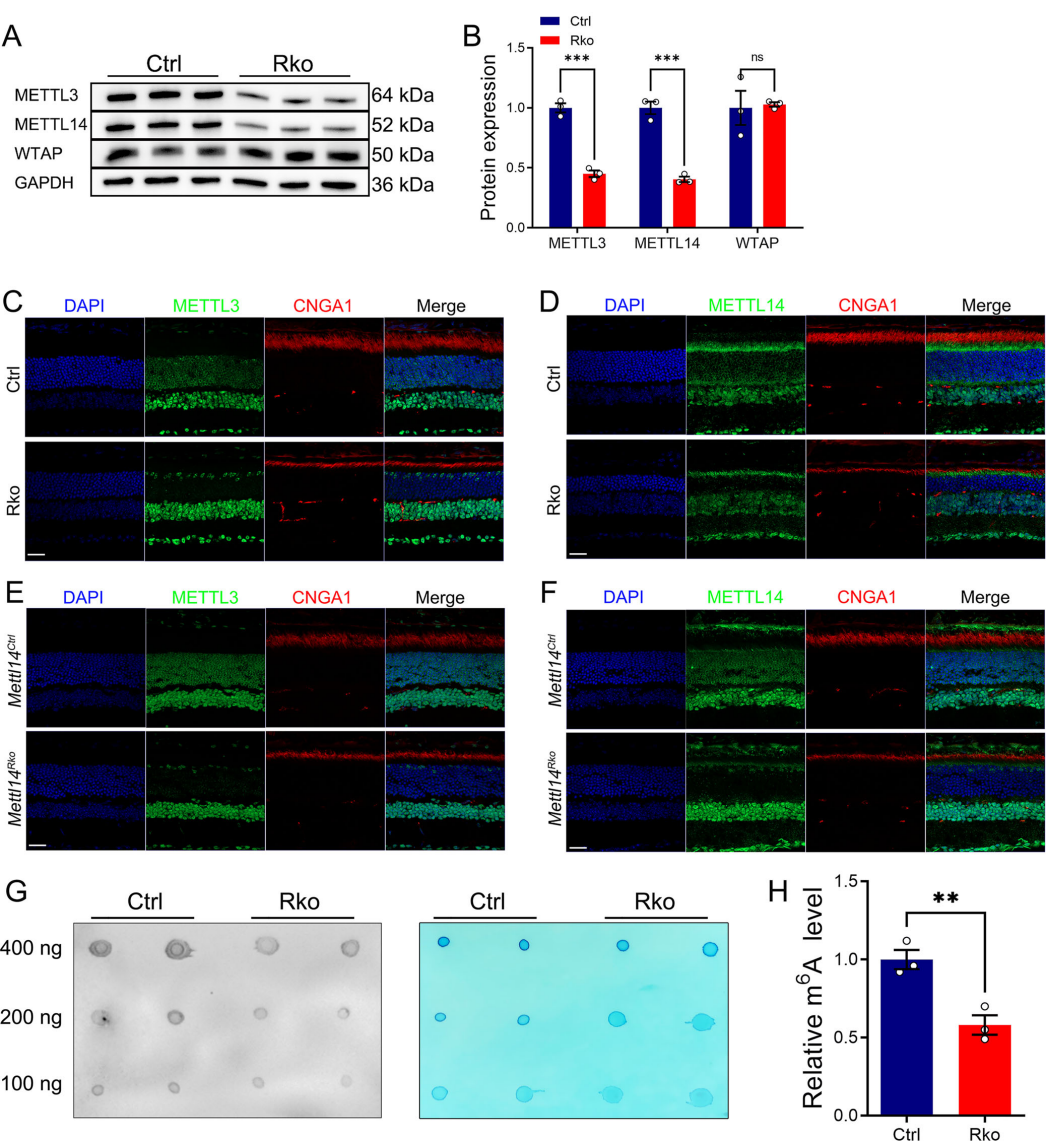
Loss of *Mettl3* led to diminished expression level of m^6^A methyltransferase complex subunit METTL14 and m^6^A modification levels. (**A**) Western blot analysis of m^6^A methyltransferase complex subunits METTL3, METTL14, and WTAP in the retina of three-month-old Ctrl and RKO mice, with GAPDH as a loading control. (**B**) Quantification of METTL3, METTL14, and WTAP protein expression levels and inter-group difference analysis. Number of samples per group, *n* = 3. Significance levels: ***P < 0.001; ns represent no significance. (**C**, **D**) Immunofluorescence staining of frozen retinal sections from Ctrl and RKO mice. *Green* represents METTL3 (**C**) and METTL14 (**D**), marking their expression locations in the retina. *Red* represents CNGA1 labeling the outer segments. *Scale bars:* 30 µm. (**E**, **F**) Immunofluorescence staining of frozen retinal sections from *Mettl14^Ctrl^* and *Mettl14^RKO^* mice. Green fluorescence indicates the expression of METTL3 (**E**) and METTL14 (**F**) in the retina. Red fluorescence marks CNGA1 in the outer segments. *Scale bar:* 30 µm. None; Dot blot images showing m^6^A methylation levels in the retina of six-month-old mice, with the left image representing spots captured by incubation with an m^6^A antibody and the right image showing methylene blue staining for control. (**H**) Statistical analysis of the grayscale values from dot blot images obtained by incubation with the m^6^A antibody. Number of samples per group, *n* = 3, **P < 0.01.

METTL3 is the catalytically active subunit of the m^6^A methyltransferase complex, which is directly involved in m^6^A modification of adenosine (A).^14^^,^^45^^,^^46^ It forms a heterodimer with METTL14. METTL14 mainly plays an auxiliary role to enhance the catalytic activity of METTL3.^14^ In addition, METTL3 interacts with other auxiliary proteins (WTAP, VIRMA, etc.) to enhance the stability of the complex.^47^^,^^48^ To investigate the expression of METTL3 and METTL14 in the retina of RKO mice, We first performed Western blot experiments and found that METTL3 and METTL14 proteins were down-regulated by 55% and 60%, respectively, in the retina of RKO mice (Figs. 5 A, 5B). The expression level of another auxiliary protein WTAP was not affected by METTL3 deletion (Fig. 4A, 4B), which may be due to the fact that the binding between WTAP and METTL3 is transient or temporary.^49^ We also further verified this result by immunofluorescence staining analysis and found that, similar to that of METTL3 (Fig. 5C), the expression level of METTL14 was significantly reduced in the ONL of *Mettl3* RKO mice (Fig. 5D). Similarly, the expression levels of METTL3 and METTL14 were also significantly reduced in the ONL of *Mettl14* RKO mice (Figs. 5E, 5F). Next, we explored the changes of m^6^A modification levels in the retina of RKO mice by m^6^A Dot blot experiments (Fig. 5G), and found that the m^6^A modification level was downregulated by about 45% in RKO mice (Fig. 5H), indicating that deletion of *Mettl3* resulted in a direct decrease in m^6^A modification levels in the retina. Given that rod cells constitute approximately 60% of the total retinal cells, the overall reduction in m^6^A modification levels in RKO was significant.

### Multi-Omics Analysis Identifies Potential Target Genes for *Mettl3* in Retinal Rod Cells

**Figure 6.**
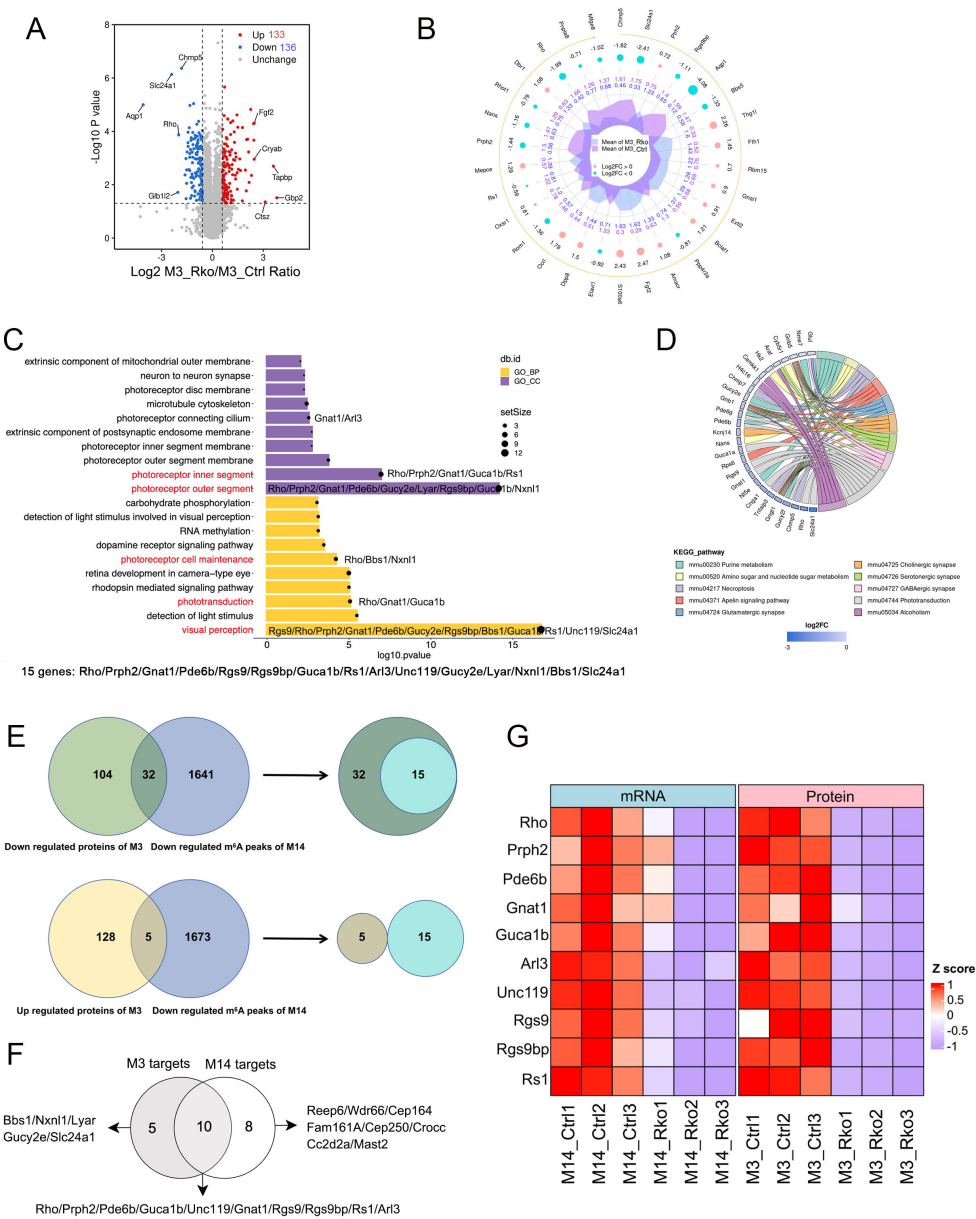
Proteomic data analysis of 12-week-old Ctrl and RKO mice. (**A**) Volcano plot of the proteomics, with values representing changes in RKO relative to Ctrl; proteins upregulated are marked in *red*, downregulated in *blue*, and those without significant differences in *gray*. (**B**) Dynamic map of protein changes in proteomics from RKO compared to Ctrl; upregulated proteins are indicated with *pink circles*, downregulated proteins with *blue circles*, *purple values* represent relative expression levels of Ctrl proteins, and *blue values* represent those of RKO proteins. (**C**) Gene ontology (GO) functional enrichment analysis of the differentially expressed proteins identified in the proteome. (**D**) Cripolt diagram showing the distribution of differentially expressed proteins among the top 10 KEGG pathways, with cripolt illustrating the detailed relationship between expression levels of proteins (left semicircle) and their enriched KEGG pathways (right semicircle). Genes are connected to their annotated terms by colored ribbons. (**E**) Venn diagram of METTL3 proteomics and METTL14 MeRIP-seq. (**F**) Venn diagram of METTL3 targets genes and METTL14 targets genes. (**G**) Construct heatmaps for the selected 10 genes based on their expression in METTL14 transcriptomics and METTL3 proteomics. M_14 represents METTL14, M_3 represents METTL3. M3 represents METTL3, M14 represents METTL14.

A reduction in the level of reduced m^6^A modification results in diminished binding affinity for reader proteins, which affects various biological processes such as mRNA export, stability, and translation efficiency. To investigate the molecular mechanisms underlying retinal degeneration after *Mettl3* deletion, we performed proteomic analysis on 2.5-month-old control and RKO mice, which started to exhibit retinal degeneration (Fig. S7). A total of 133 up-regulated proteins and 136 down-regulated proteins were identified in RKO mice compared with controls (foldchange >1.5 or < 1/1.5; *P* < 0.05) (Fig. 6A). The top 30 significant differentially expressed proteins (DEPs) are shown as radar plots (Fig. 6B). Gene ontology (GO) enrichment analysis of the 269 differentially expressed genes revealed that the majority of these genes are associated with photoreceptor outer and inner segments, as well as visual perception pathways (Fig. 6C, highlighted in red). By integrating the genes highlighted in the pathways, we identified a total of 15 genes closely related to photoreceptor function (Fig. 6C, bottom). The reference DEPs mapped to KEGG enrichment analysis pathways, including several key genes within photoreceptors, was illustrated (Fig. 6D). Using previous m^6^A sequencing data in *Mettl14* rod specific knockout mice,^35^ we compared genes with downregulated m^6^A modification with genes downregulated in protein expression after *Mettl3* knockout and identified a group of 32 genes. Similarly, we found five intersecting genes between downregulated m^6^A-modified genes and protein upregulated after *Mettl3* knockout (Fig. 6E). Further analysis revealed that all 15 genes identified in Figure 6C are among the 32 genes downregulated in m^6^A modification and downregulated in protein expression after *Mettl3* knockout (Fig. 6E). A majority of these 15 target genes of METTL3 overlapped with 18 target genes of METTL14 as previously identified,^35^ and the overlapped 10 genes (*Rho, Prph2, Pde6b, Gnat1, Guca1b, Arl3, Unc119, RGS9, Rgs9bp, Rs1*) were assigned as the most probable METTL3 target genes (Fig. 6F). Heatmap analysis of these 10 selected genes showed a consistent downregulation trend in both mRNA levels in METTL14 transcriptome sequencing and protein levels in METTL3 proteome sequencing (Fig. 6G).

### *Mettl3* Regulates the Expression of Multiple Phototransduction-Related Proteins

**Figure 7.**
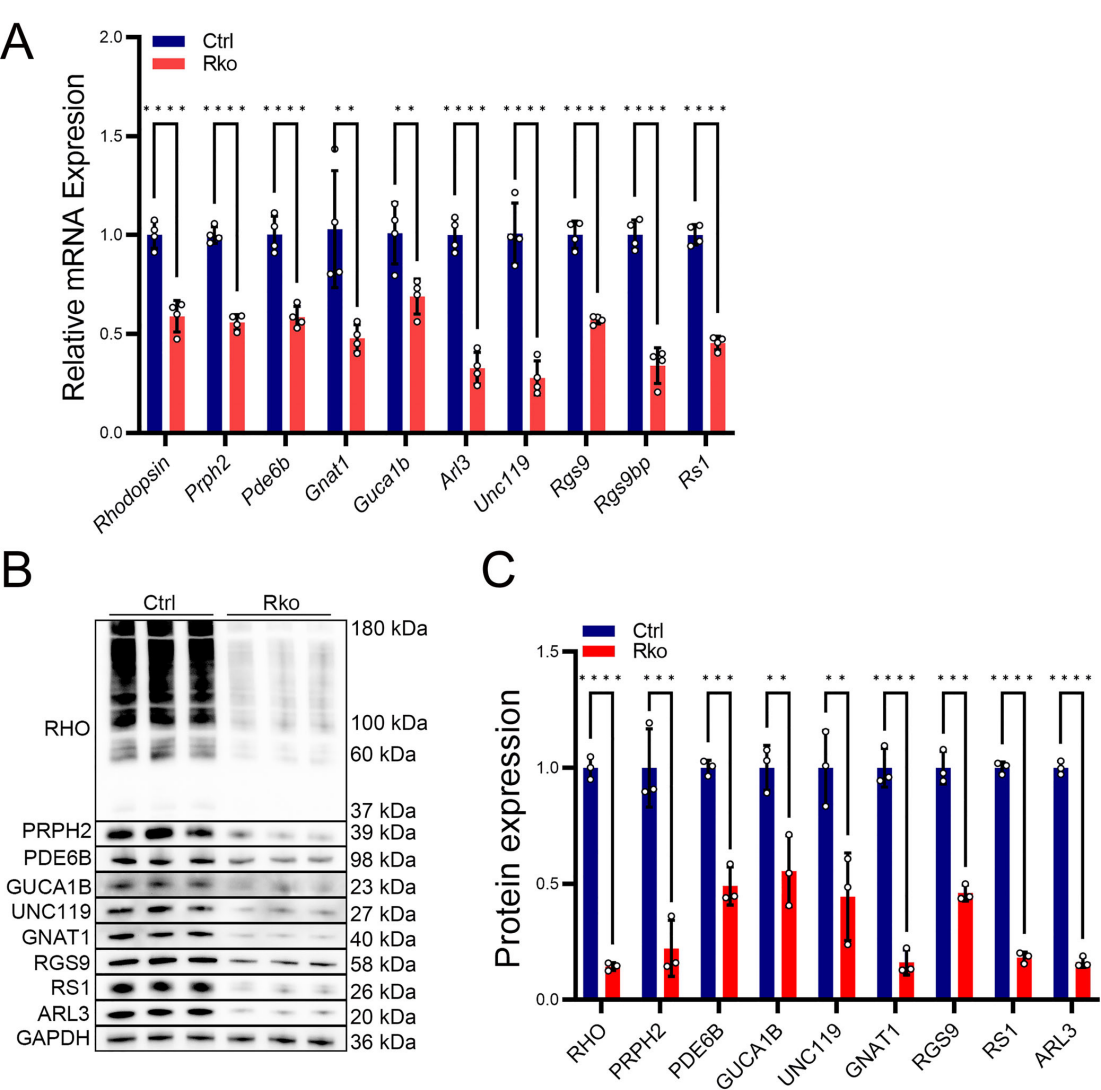
Validation of the expression change of selected downstream genes. (**A**) We extracted RNA from the retinal tissue of 2.5-month-old Ctrl and RKO mice and verified the mRNA expression levels of the target genes using RT-qPCR experiments. Statistical analysis revealed a significant downregulation in the mRNA expression levels of the 10 target genes. Number of samples per group, *n* = 4. (**B**) We validated the protein expression levels of the target genes in 2.5-month-old Ctrl and RKO mice using Western blot experiments. (C) Using ImageJ software, we calculated the grayscale values of the Western blot images and performed statistical analysis to determine the expression levels of each protein. Number of samples per group, *n* = 3. **P < 0.01, ***P < 0.001, ****P < 0.0001.

To determine whether the above mentioned 10 candidate genes we identified were target genes of *Mettl3*, we designed a series of validation experiments. RT-qPCR experiments revealed that the expression levels of mRNA of all 10 genes were significantly down-regulated (Fig. 7A), suggesting that deletion of *Mettl3* may have affected the stability of the mRNAs of the target genes. Furthermore, we performed protein expression assays. Since we were unable to find a suitable primary antibody to RGS9BP, we performed western blot experiments on the protein expression of the other nine genes excluding *Rgs9bp*. Protein expression levels were also decreased for all 9 proteins except RGS9BP (Figs. 7B, 7C). The aforementioned results indicated that loss of *Mettl3* leads to reduction in m^6^A modification levels of target genes, thereby decreasing the binding of reader proteins responsible for mRNA stability and translation efficiency. Consequently, this results in decreased mRNA and protein expression levels of the target genes, which contribute to photoreceptor degeneration.

## Discussion

In this study, a series of phenotypic experiments on *Mettl3* RKO and CKO mouse models revealed that *Mettl3* deletion resulted in retinal degeneration phenotypic features such as diminished visual function and photoreceptor degeneration. Further mechanistic probing suggested that *Mettl3* may trigger photoreceptor degeneration by regulating the expression of several key genes related to vision formation in the retina. Although previous work has demonstrated that *Mettl14* is indispensable for both photoreceptor cell function and survival, elucidation of the function of *Mettl3*, as the only catalytically active key protein in the methyltransferase complex, in photoreceptor cells can further stress the importance of m^6^A modification in visual maintenance and provide theoretical support for subsequent treatment of retinal diseases.

After specific deletion of *Mettl3* in rod cells, western blot and immunohistochemistry demonstrated a significant reduction in METTL14 protein expression in these cells (Figs. 5A–D), suggesting close interaction of METTL3 and METTL14. This result is analogous to the downregulation of METTL3 expression observed in *Mettl14* deletion in rod cells.^35^ Meanwhile, dot blot analysis revealed that m^6^A modification in the retina of RKO mice was reduced by approximately 45% in comparison to the overall level observed in the control group (Fig. 5 G, 5H). Subsequent proteomic analysis combined with m^6^A sequencing and RNA-seq from previous studies^35^ identified ten potential target genes likely regulated by METTL3-mediated m^6^A modification (Figs. 6E, 6F). Furthermore, RT-qPCR and western blot experiments confirmed that these target genes were down-regulated at both the mRNA and protein levels (Figs. 7A–C). Several readers such as YTHDC1, YTHDC2, YTHDF1, YTHDF2, YTHDF3, IGF2BP2, and HNRNPA2B1 are highly expressed in rods/cones. In rod and cone photoreceptors, m6A-modified mRNA can be recognized by one or multiple readers, leading to distinct functional outcomes. The observed downregulation of mRNA targets upon *Mettl3* deletion suggests that these readers may play a key role in stabilizing or enhancing the translation of these transcripts in photoreceptors. This may be attributed to the reduced binding of m^6^A reader proteins, which are responsible for regulating the stability and translational efficiency of mRNA, following the reduction of *Mettl3*-mediated m^6^A modification.

Phototransduction represents a pivotal process within the visual system, whereby light signals are converted into electrical signals. This process is orchestrated by a complex network of proteins and signaling molecules, with the precise regulation between them ensuring the smooth progression of the phototransduction process.^50^ Five of the target genes identified belongs to members of the phototransduction pathway, namely *Rho*, *Pde6b*, *Gnat1*, *Rgs9*, and *Guca1b*. Mutations in the aforementioned phototransduction-related genes have been previously documented in the literature to induce severe retinal degenerative diseases. For instance, *RHO* mutations primarily result in protein misfolding, which subsequently induces endoplasmic reticulum stress and ultimately leads to the demise of rod cells, thereby causing retinal degeneration.^51^^,^^52^ Mutations in *PDE6B*, which encodes the β-subunit of PDE6, lead to disorders such as arRP.^53^ The Pde6b mutant mice (*Pde6b^rd1^* and *Pde6b^rd10^*) exhibit photoreceptor degeneration, among other symptoms.^54^^,^^55^ Mutations in *GNAT1* result in the inability of the rod cells to activate the downstream phosphodiesterase (PDE6), which affects the degradation of cGMP. This ultimately leads to the death of the rod cells and retinal degeneration. *Gnat1*-mutated mouse models exhibit signs of night blindness and progressive degeneration of the rods over time.^56^ Mutations in *RGS9*, which terminates light signaling by accelerating the activity of the G protein GTPase, lead to abnormalities in light signaling, which in turn cause retinitis pigmentosa and congenital stationary night blindness.^57^ Moreover, mutations in *GUCA1B*, which encodes a calcium-binding protein and activates the photoreceptor guanylate cyclase, lead to retinitis pigmentosa, cone dystrophy and cone-rod dystrophy 2.^58^^–^^60^

The remaining target genes also play critical roles in rod cells. *PRPH2* and *RS1* are essential for the maintenance of the normal structure of the retina, and mutations in either of them can lead to a variety of retinal degenerative diseases, including RP.^61^^,^^62^ *UNC119* is mainly involved in regulating the function of photoreceptor cells in the retina, and mutations in *UNC119* can lead to neurodegenerative disorders, including retinal diseases.^63^ *RGS9BP* acts as a binding protein for RGS9, stabilizing the structure of RGS9 and enhancing the regulation of the G-protein signaling pathway by RGS9.^64^ Mutations in *RGS9BP* lead to an inherited disease known as Brady′s vision, in which patients suffer from reduced visual adaptation to sudden changes in light and are subjected to significant impairment of visual function.^65^ ARL3 regulates the transport of prenylated proteins and the cilia of the rod OS,^66^ and mutations in *ARL3* lead to diseases such as retinitis pigmentosa and congenital black blindness.^67^

The present study demonstrates that *Mettl3*-mediated m^6^A modification can regulate the mRNA and protein expression levels of several target genes in rod cells. This has important effects on the phototransduction process, rod cell structure and internal protein transport, thus affecting the function and survival of photoreceptor cells. Our study contributes to the understanding of the function and mechanism of action of *Mettl3*, a core member of the writer complex, in photoreceptor cells. This complements the regulatory mechanism of m^6^A modification in photoreceptor cells and sheds light on potential application of m^6^A modification in the therapeutic development of retinal degenerative diseases.

## Supplementary Material

Supplement 1

## References

[1] Jiang X, Liu B, Nie Z, et al. The role of m6A modification in the biological functions and diseases. *Signal Transduct Target Ther*. 2021; 6: 74. 33611339 10.1038/s41392-020-00450-x PMC7897327

[2] Oerum S, Meynier V, Catala M, Tisné C. A comprehensive review of m6A/m6Am RNA methyltransferase structures. *Nucleic Acids Res*. 2021; 49: 7239–7255. 34023900 10.1093/nar/gkab378 PMC8287941

[3] Zheng G, Dahl JA, Niu Y, et al. ALKBH5 is a mammalian RNA demethylase that impacts RNA metabolism and mouse fertility. *Mol Cell*. 2013; 49: 18–29. 23177736 10.1016/j.molcel.2012.10.015 PMC3646334

[4] Zhao Y, Shi Y, Shen H, Xie W. m(6)A-binding proteins: the emerging crucial performers in epigenetics. *J Hematol Oncol*. 2020; 13: 35. 32276589 10.1186/s13045-020-00872-8 PMC7146974

[5] Geula S, Moshitch-Moshkovitz S, Dominissini D, et al. Stem cells. m6A mRNA methylation facilitates resolution of naïve pluripotency toward differentiation. *Science*. 2015; 347(6225): 1002–1006. 25569111 10.1126/science.1261417

[6] Batista PJ, Molinie B, Wang J, et al. m(6)A RNA modification controls cell fate transition in mammalian embryonic stem cells. *Cell Stem Cell*. 2014; 15: 707–719. 25456834 10.1016/j.stem.2014.09.019 PMC4278749

[7] Fustin JM, Doi M, Yamaguchi Y, et al. RNA-methylation-dependent RNA processing controls the speed of the circadian clock. *Cell*. 2013; 155: 793–806. 24209618 10.1016/j.cell.2013.10.026

[8] Winkler R, Gillis E, Lasman L, et al. m(6)A modification controls the innate immune response to infection by targeting type I interferons. *Nat Immunol*. 2019; 20: 173–182. 30559377 10.1038/s41590-018-0275-z

[9] Chen M, Wong CM. The emerging roles of N6-methyladenosine (m6A) deregulation in liver carcinogenesis. *Mol Cancer*. 2020; 19: 44. 32111216 10.1186/s12943-020-01172-y PMC7047367

[10] He L, Li H, Wu A, Peng Y, Shu G, Yin G. Functions of N6-methyladenosine and its role in cancer. *Mol Cancer*. 2019; 18: 176. 31801551 10.1186/s12943-019-1109-9 PMC6892141

[11] Yoon KJ, Ringeling FR, Vissers C, et al. Temporal control of mammalian cortical neurogenesis by m(6)A methylation. *Cell*. 2017; 171: 877–889.e17. 28965759 10.1016/j.cell.2017.09.003 PMC5679435

[12] Zhang C, Chen Y, Sun B, et al. m(6)A modulates haematopoietic stem and progenitor cell specification. *Nature*. 2017; 549(7671): 273–276. 28869969 10.1038/nature23883

[13] Huang H, Weng H, Chen J. The biogenesis and precise control of RNA m(6)A methylation. *Trends Genet*. 2020; 36: 44–52. 31810533 10.1016/j.tig.2019.10.011 PMC6925345

[14] Liu J, Yue Y, Han D, et al. A METTL3-METTL14 complex mediates mammalian nuclear RNA N6-adenosine methylation. *Nat Chem Biol*. 2014; 10: 93–95. 24316715 10.1038/nchembio.1432 PMC3911877

[15] Chen M, Wei L, Law CT, et al. RNA N6-methyladenosine methyltransferase-like 3 promotes liver cancer progression through YTHDF2-dependent posttranscriptional silencing of SOCS2. *Hepatology*. 2018; 67: 2254–2270. 29171881 10.1002/hep.29683

[16] Lin S, Choe J, Du P, Triboulet R, Gregory RI. The m(6)A methyltransferase METTL3 promotes translation in human cancer cells. *Mol Cell*. 2016; 62: 335–345. 27117702 10.1016/j.molcel.2016.03.021 PMC4860043

[17] Barbieri I, Tzelepis K, Pandolfini L, et al. Promoter-bound METTL3 maintains myeloid leukaemia by m(6)A-dependent translation control. *Nature*. 2017; 552(7683): 126–131. 29186125 10.1038/nature24678 PMC6217924

[18] Vu LP, Pickering BF, Cheng Y, et al. The N(6)-methyladenosine (m(6)A)-forming enzyme METTL3 controls myeloid differentiation of normal hematopoietic and leukemia cells. *Nat Med*. 2017; 23: 1369–1376. 28920958 10.1038/nm.4416 PMC5677536

[19] Cui Q, Shi H, Ye P, et al. m(6)A RNA methylation regulates the self-renewal and tumorigenesis of glioblastoma stem cells. *Cell Rep*. 2017; 18: 2622–2634. 28297667 10.1016/j.celrep.2017.02.059 PMC5479356

[20] Visvanathan A, Patil V, Arora A, et al. Essential role of METTL3-mediated m(6)A modification in glioma stem-like cells maintenance and radioresistance. *Oncogene*. 2018; 37: 522–533. 28991227 10.1038/onc.2017.351

[21] Wu Y, Xie L, Wang M, et al. Mettl3-mediated m(6)A RNA methylation regulates the fate of bone marrow mesenchymal stem cells and osteoporosis. *Nat Commun*. 2018; 9(1): 4772. 30429466 10.1038/s41467-018-06898-4 PMC6235890

[22] Dorn LE, Lasman L, Chen J, et al. The N(6)-methyladenosine mRNA methylase METTL3 controls cardiac homeostasis and hypertrophy. *Circulation*. 2019; 139: 533–545. 30586742 10.1161/CIRCULATIONAHA.118.036146 PMC6340720

[23] Xiong J, He J, Zhu J, et al. Lactylation-driven METTL3-mediated RNA m(6)A modification promotes immunosuppression of tumor-infiltrating myeloid cells. *Mol Cell*. 2022; 82: 1660–1677.e10. 35320754 10.1016/j.molcel.2022.02.033

[24] Hartong DT, Berson EL, Dryja TP. Retinitis pigmentosa. *Lancet*. 2006; 368(9549): 1795–1809. 17113430 10.1016/S0140-6736(06)69740-7

[25] Wright AF, Chakarova CF, Abd El-Aziz MM, Bhattacharya SS. Photoreceptor degeneration: genetic and mechanistic dissection of a complex trait. *Nat Rev Genet*. 2010; 11: 273–284. 20212494 10.1038/nrg2717

[26] Daiger SP, Sullivan LS, Bowne SJ. Genes and mutations causing retinitis pigmentosa. *Clin Genet*. 2013; 84: 132–141. 23701314 10.1111/cge.12203 PMC3856531

[27] Gao FJ, Li JK, Chen H, et al. Genetic and clinical findings in a large cohort of Chinese patients with suspected retinitis pigmentosa. *Ophthalmology*. 2019; 126: 1549–1556. 31054281 10.1016/j.ophtha.2019.04.038

[28] Yang M, Li S, Liu W, et al. The ER membrane protein complex subunit Emc3 controls angiogenesis via the FZD4/WNT signaling axis. *Sci China Life Sci*. 2021; 64: 1868–1883. 34128175 10.1007/s11427-021-1941-7

[29] Jiang L, Dai C, Wei Y, et al. Identification of LRRC46 as a novel candidate gene for high myopia. *Sci China Life Sci*. 2024; 67: 1941–1956. 38874710 10.1007/s11427-024-2583-6

[30] Bainbridge JW, Smith AJ, Barker SS, et al. Effect of gene therapy on visual function in Leber′s congenital amaurosis. *N Engl J Med*. 2008; 358: 2231–2239. 18441371 10.1056/NEJMoa0802268

[31] Chung DC, Traboulsi EI. Leber congenital amaurosis: clinical correlations with genotypes, gene therapy trials update, and future directions. *J AAPOS*. 2009; 13: 587–592. 20006823 10.1016/j.jaapos.2009.10.004

[32] Scholl HP, Strauss RW, Singh MS, et al. Emerging therapies for inherited retinal degeneration. *Sci Transl Med*. 2016; 8(368): 368rv6. 10.1126/scitranslmed.aaf2838 27928030

[33] Gagliardi G, Ben M′Barek K, Goureau O. Photoreceptor cell replacement in macular degeneration and retinitis pigmentosa: A pluripotent stem cell-based approach. *Prog Retin Eye Res*. 2019; 71: 1–25. 30885665 10.1016/j.preteyeres.2019.03.001

[34] Farrar GJ, Carrigan M, Dockery A, et al. Toward an elucidation of the molecular genetics of inherited retinal degenerations. *Hum Mol Genet*. 2017; 26(R1): R2–R11. 28510639 10.1093/hmg/ddx185 PMC5886474

[35] Yang Y, Shuai P, Li X, et al. Mettl14-mediated m6A modification is essential for visual function and retinal photoreceptor survival. *BMC Biol*. 2022; 20: 140. 35698136 10.1186/s12915-022-01335-x PMC9195452

[36] Li S, Chen D, Sauvé Y, McCandless J, Chen YJ, Chen CK. Rhodopsin-iCre transgenic mouse line for Cre-mediated rod-specific gene targeting. *Genesis*. 2005; 41: 73–80. 15682388 10.1002/gene.20097

[37] Le Y Z, Ash JD, Al-Ubaidi MR, Chen Y, Ma JX, Anderson RE. Targeted expression of Cre recombinase to cone photoreceptors in transgenic mice. *Mol Vis*. 2004; 10: 1011–1018. 15635292

[38] Madisen L, Zwingman TA, Sunkin SM, et al. A robust and high-throughput Cre reporting and characterization system for the whole mouse brain. *Nat Neurosci*. 2010; 13: 133–140. 20023653 10.1038/nn.2467 PMC2840225

[39] Pearring JN, Salinas RY, Baker SA, Arshavsky VY. Protein sorting, targeting and trafficking in photoreceptor cells. *Prog Retin Eye Res*. 2013; 36: 24–51. 23562855 10.1016/j.preteyeres.2013.03.002 PMC3759535

[40] Booij JC, Florijn RJ, ten Brink JB, et al. Identification of mutations in the AIPL1, CRB1, GUCY2D, RPE65, and RPGRIP1 genes in patients with juvenile retinitis pigmentosa. *J Med Genet*. 2005; 42(11): e67. 16272259 10.1136/jmg.2005.035121 PMC1735944

[41] Bringmann A, Pannicke T, Grosche J, et al. Müller cells in the healthy and diseased retina. *Prog Retin Eye Res*. 2006; 25: 397–424. 16839797 10.1016/j.preteyeres.2006.05.003

[42] Vecino E, Rodriguez FD, Ruzafa N, Pereiro X, Sharma SC. Glia-neuron interactions in the mammalian retina. *Prog Retin Eye Res*. 2016; 51: 1–40. 26113209 10.1016/j.preteyeres.2015.06.003

[43] Nathans J. The evolution and physiology of human color vision: insights from molecular genetic studies of visual pigments. *Neuron*. 1999; 24: 299–312. 10571225 10.1016/s0896-6273(00)80845-4

[44] Schnapf JL, Kraft TW, Baylor DA. Spectral sensitivity of human cone photoreceptors. *Nature*. 1987; 325(6103): 439–441. 3808045 10.1038/325439a0

[45] Wang P, Doxtader KA, Nam Y. Structural basis for cooperative function of Mettl3 and Mettl14 methyltransferases. *Mol Cell*. 2016; 63: 306–317. 27373337 10.1016/j.molcel.2016.05.041 PMC4958592

[46] Dominissini D, Moshitch-Moshkovitz S, Schwartz S, et al. Topology of the human and mouse m6A RNA methylomes revealed by m6A-seq. *Nature*. 2012; 485(7397): 201–206. 22575960 10.1038/nature11112

[47] Ping XL, Sun BF, Wang L, et al. Mammalian WTAP is a regulatory subunit of the RNA N6-methyladenosine methyltransferase. *Cell Res*. 2014; 24: 177–189. 24407421 10.1038/cr.2014.3 PMC3915904

[48] Schwartz S, Agarwala SD, Mumbach MR, et al. High-resolution mapping reveals a conserved, widespread, dynamic mRNA methylation program in yeast meiosis. *Cell*. 2013; 155: 1409–1421. 24269006 10.1016/j.cell.2013.10.047 PMC3956118

[49] Sorci M, Ianniello Z, Cruciani S, et al. METTL3 regulates WTAP protein homeostasis. *Cell Death Dis*. 2018; 9: 796. 30038300 10.1038/s41419-018-0843-z PMC6056540

[50] Arshavsky VY, Lamb TD, Pugh EN Jr. G proteins and phototransduction. *Annu Rev Physiol*. 2002; 64: 153–187. 11826267 10.1146/annurev.physiol.64.082701.102229

[51] Mendes HF, van der Spuy J, Chapple JP, Cheetham ME. Mechanisms of cell death in rhodopsin retinitis pigmentosa: implications for therapy. *Trends Mol Med*. 2005; 11: 177–185. 15823756 10.1016/j.molmed.2005.02.007

[52] Athanasiou D, Aguila M, Bellingham J, et al. The molecular and cellular basis of rhodopsin retinitis pigmentosa reveals potential strategies for therapy. *Prog Retin Eye Res*. 2018; 62: 1–23. 29042326 10.1016/j.preteyeres.2017.10.002 PMC5779616

[53] McLaughlin ME, Sandberg MA, Berson EL, Dryja TP. Recessive mutations in the gene encoding the beta-subunit of rod phosphodiesterase in patients with retinitis pigmentosa. *Nat Genet*. 1993; 4: 130–134. 8394174 10.1038/ng0693-130

[54] Pittler SJ, Baehr W. Identification of a nonsense mutation in the rod photoreceptor cGMP phosphodiesterase beta-subunit gene of the rd mouse. *Proc Natl Acad Sci USA*. 1991; 88: 8322–8326. 1656438 10.1073/pnas.88.19.8322 PMC52500

[55] Chang B, Hawes NL, Hurd RE, Davisson MT, Nusinowitz S, Heckenlively JR. Retinal degeneration mutants in the mouse. *Vision Res*. 2002; 42: 517–525. 11853768 10.1016/s0042-6989(01)00146-8

[56] Calvert PD, Krasnoperova NV, Lyubarsky AL, et al. Phototransduction in transgenic mice after targeted deletion of the rod transducin alpha -subunit. *Proc Natl Acad Sci USA*. 2000; 97: 13913–13918. 11095744 10.1073/pnas.250478897 PMC17675

[57] Jayaraman M, Zhou H, Jia L, Cain MD, Blumer KJ. R9AP and R7BP: traffic cops for the RGS7 family in phototransduction and neuronal GPCR signaling. *Trends Pharmacol Sci*. 2009; 30: 17–24. 19042037 10.1016/j.tips.2008.10.002 PMC2776672

[58] Dizhoor AM . Regulation of cGMP synthesis in photoreceptors: role in signal transduction and congenital diseases of the retina. *Cell Signal*. 2000; 12(11–12): 711–719. 11152956 10.1016/s0898-6568(00)00134-0

[59] Kitiratschky VB, Glöckner CJ, Kohl S. Mutation screening of the GUCA1B gene in patients with autosomal dominant cone and cone rod dystrophy. *Ophthalmic Genet*. 2011; 32: 151–155. 21405999 10.3109/13816810.2011.559650

[60] Castori M, Valente EM, Clementi M, et al. A novel locus for autosomal dominant cone and cone-rod dystrophies maps to the 6p gene cluster of retinal dystrophies. *Invest Ophthalmol Vis Sci*. 2005; 46: 3539–3544. 16186331 10.1167/iovs.05-0331

[61] Boon CJ, den Hollander AI, Hoyng CB, Cremers FP, Klevering BJ, Keunen JE. The spectrum of retinal dystrophies caused by mutations in the peripherin/RDS gene. *Prog Retin Eye Res*. 2008; 27: 213–235. 18328765 10.1016/j.preteyeres.2008.01.002

[62] Tantri A, Vrabec TR, Cu-Unjieng A, Frost A, Annesley WH Jr., Donoso LA. X-linked retinoschisis: a clinical and molecular genetic review. *Surv Ophthalmol*. 2004; 49: 214–230. 14998693 10.1016/j.survophthal.2003.12.007

[63] Zhang H, Constantine R, Vorobiev S, et al. UNC119 is required for G protein trafficking in sensory neurons. *Nat Neurosci*. 2011; 14: 874–880. 21642972 10.1038/nn.2835 PMC3178889

[64] Martemyanov KA, Yoo PJ, Skiba NP, Arshavsky VY. R7BP, a novel neuronal protein interacting with RGS proteins of the R7 family. *J Biol Chem*. 2005; 280: 5133–5136. 15632198 10.1074/jbc.C400596200

[65] Nishiguchi KM, Sandberg MA, Kooijman AC, et al. Defects in RGS9 or its anchor protein R9AP in patients with slow photoreceptor deactivation. *Nature*. 2004; 427(6969): 75–78. 14702087 10.1038/nature02170

[66] Hanke-Gogokhia C, Wu Z, Gerstner CD, Frederick JM, Zhang H, Baehr W. Arf-like protein 3 (ARL3) regulates protein trafficking and ciliogenesis in mouse photoreceptors. *J Biol Chem*. 2016; 291: 7142–7155. 26814127 10.1074/jbc.M115.710954 PMC4807295

[67] Gotthardt K, Lokaj M, Koerner C, Falk N, Giessl A, Wittinghofer A. A G-protein activation cascade from Arl13B to Arl3 and implications for ciliary targeting of lipidated proteins. *Elife*. 2015; 4: e11859. 26551564 10.7554/eLife.11859 PMC4868535

