# Supplementary material for: Mettl3-Mediated m6A Modification is Essential for Visual Function and Retinal Photoreceptor Survival
Source: Invest Ophthalmol Vis Sci. 2024 Dec 27;65(14):40. doi: 10.1167/iovs.65.14.40 (PMC11684116; doi:10.1167/iovs.65.14.40)
Supplement: Supplement 1 [file iovs-65-14-40_s001.pdf]

Mettl3-mediated m^{6}A modification is essential for visual function and retinal photoreceptor survival

Xiaoyan Jiang[1]#, Kuanxiang Sun[1]#, Yudi Fan[1]#, Qianchun Xiang[1]#, Rong Zou[1], Yeming Yang[1]*, Xianjun Zhu[1-3]*, Wenjing Liu[1]*

Supplemental data include figure S1-S7 and tabl1 S1-S2.

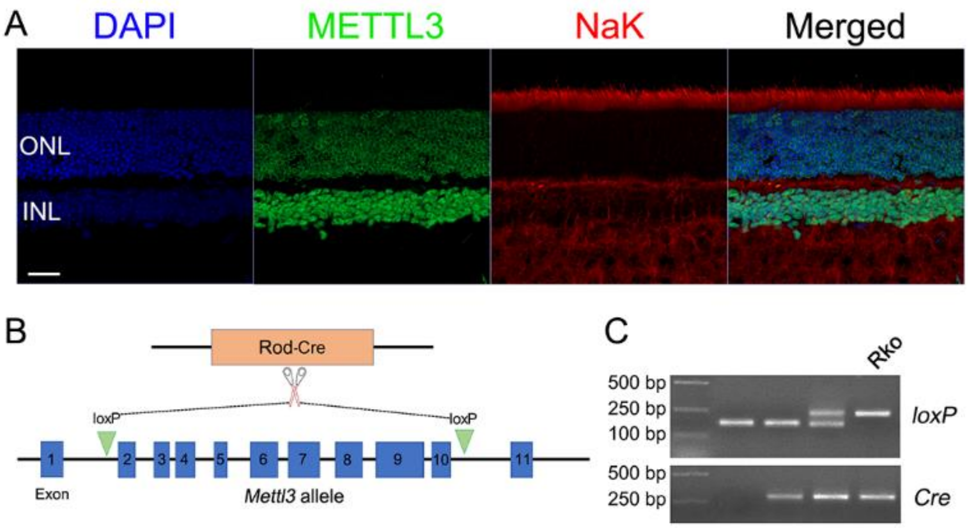

Figure S1. Generation of rod-specific *Mettl3* knockout mice. (A) Immunofluorescence staining of wild-type murine retinas revealed METTL3 protein expression in ONL and INL. Green represented METTL3 labeling, and red represents Nak ATPase labeling the inner segments. Cell nuclei were counterstained with DAPI in blue. Scale bar, 20 μm. (B) Exons 2-10 of the *Mettl3* gene were excised using the Cre-loxP recombinase system. (C) Genotyping results of *Mettl3* RKO mice during the breeding process.

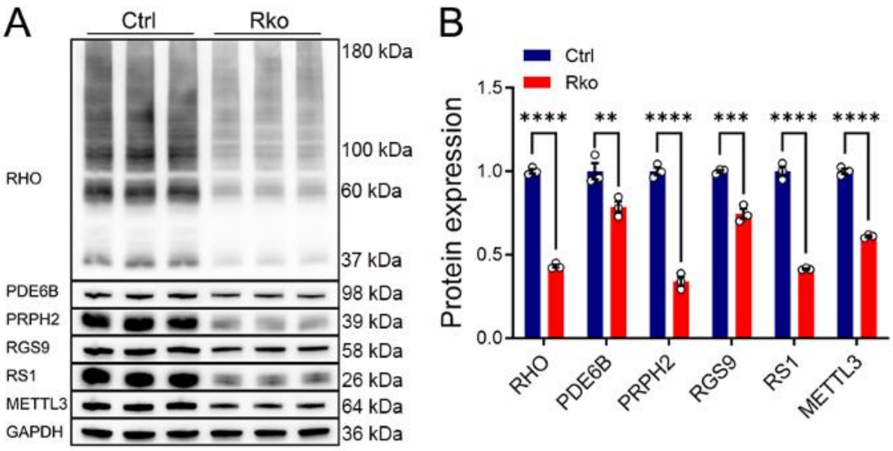

Figure S2. Downregulation of disc protein expression in *Mettl3* RKO mice at 3 months of age. (A) Western blot analysis of disc protein expression (RHO, PDE6B, PRPH2, RGS9) in the retina of Ctrl and RKO mice, with GAPDH serving as a loading control. (B) Statistical analysis of protein expression levels for RHO, PDE6B, PRPH2, and RGS9. Number of samples per group, n=3. Significance levels: ****, p < 0.0001; ***, p < 0.001; **, p < 0.01.

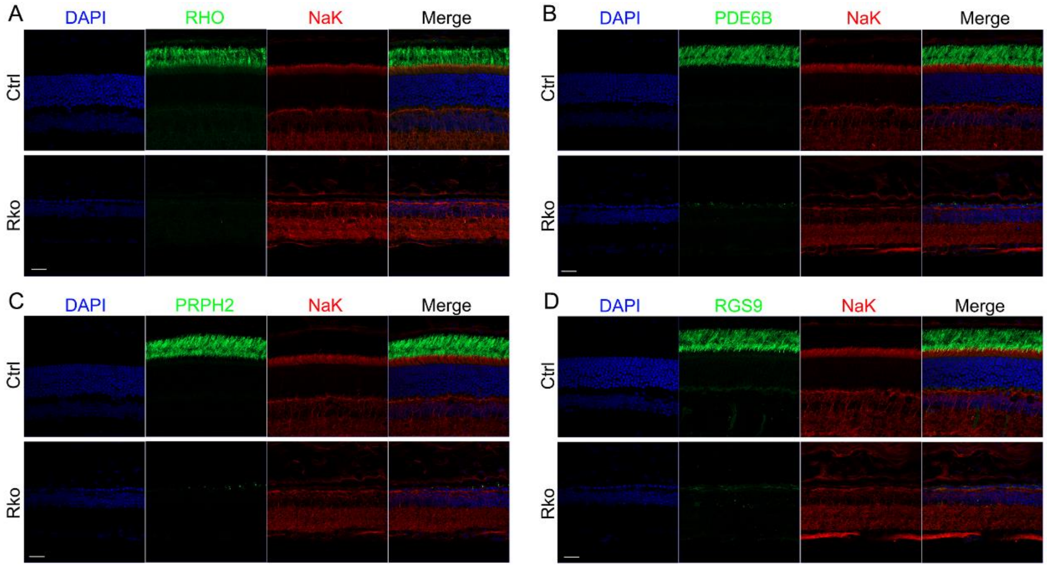

Figure S3. Immunofluorescence staining analysis of Ctrl and RKO retinas at 8 months. (A-D) Immunofluorescence staining of retinal cryosections from 8-month-old Ctrl and RKO mice. The inner segments were labeled with NaK ATPase antibody (red), while the outer segments were labeled with antibodies against RHO (A), PDE6B (B), PRPH2 (C), and RGS9 (D) (green). Cell nuclei were counterstained with DAPI (blue). Scale bar: 20 µm.

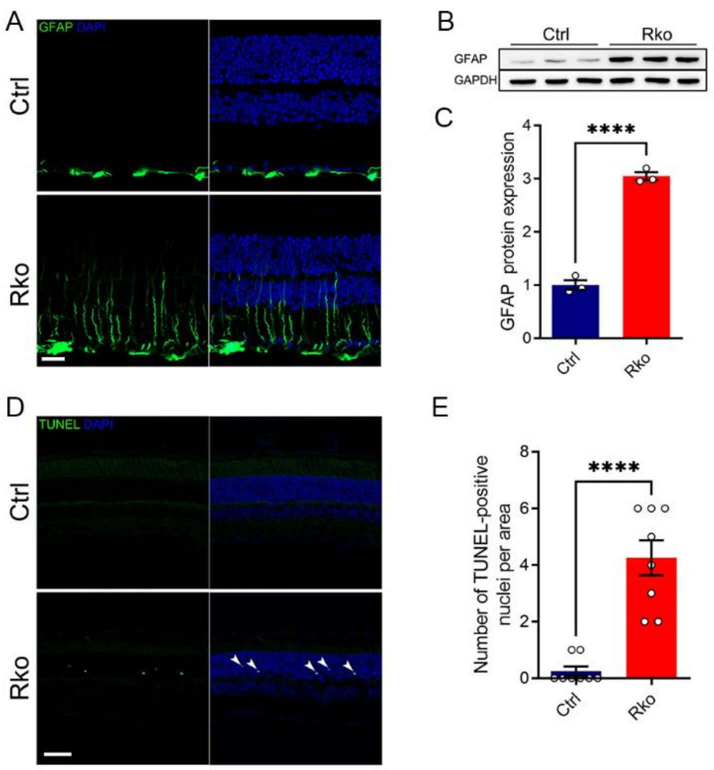

Figure S4. Proliferation of astrocytes and apoptosis in RKO mice. (A) Immunofluorescence staining of frozen retinal sections from mice, displaying GFAP in green and DAPI-stained cell nuclei in blue. Scale bar: 20 μm. (B) Quantitative analysis of GFAP in mouse retina by Western blot, with GAPDH serving as the loading control. (C) Statistical analysis of GFAP with a sample size of n=3 per group, ****: $p < 0.0001$. (D) TUNEL staining of frozen retinal sections from Ctrl and RKO mice, with green indicating TUNEL-positive nuclei and blue representing nuclei marked with DAPI. Scale bar: 50 μm. (E) Quantitative analysis of TUNEL-positive cells. Number of samples per group: n=8. ****: $p < 0.0001$.

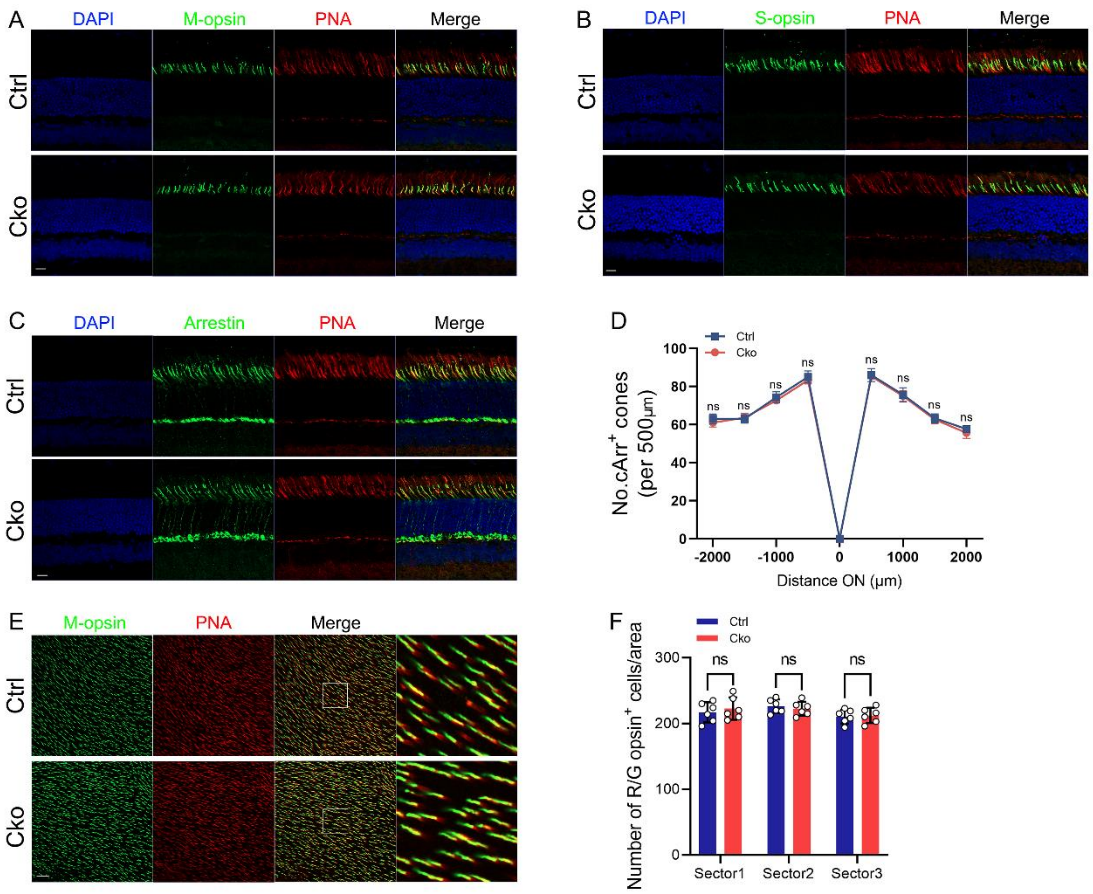

Figure S5. No visible degeneration was observed in cone photoreceptors in 3-month-old CKO mice.

(A-C) Retinal cryosections from 3-month-old mice were subjected to immunofluorescence staining to examine cone photoreceptors, with M-opsin (A), S-opsin (B), and Arrestin (C) antibodies. Co-staining with the cone-specific marker PNA (red) was performed, and DAPI (blue) was used to label nuclei. No signs of cone photoreceptor degeneration were detected at this age. Scale bar, 25 μm. (D) The number of Arrestin-positive cone photoreceptors was quantified in both Ctrl and CKO retinas at 500 μm intervals. No significant difference between the groups was observed (ns: $P > 0.05$). (E-F) Retinal whole-mounts from Ctrl and CKO mice were immunostained with M-opsin (green) and PNA (red). Quantitative analysis revealed no significant reduction in cone photoreceptor numbers in CKO retinas compared to Ctrl. Scale bar, 25 μm. Statistical comparison showed no significant differences (ns: $P > 0.05$).

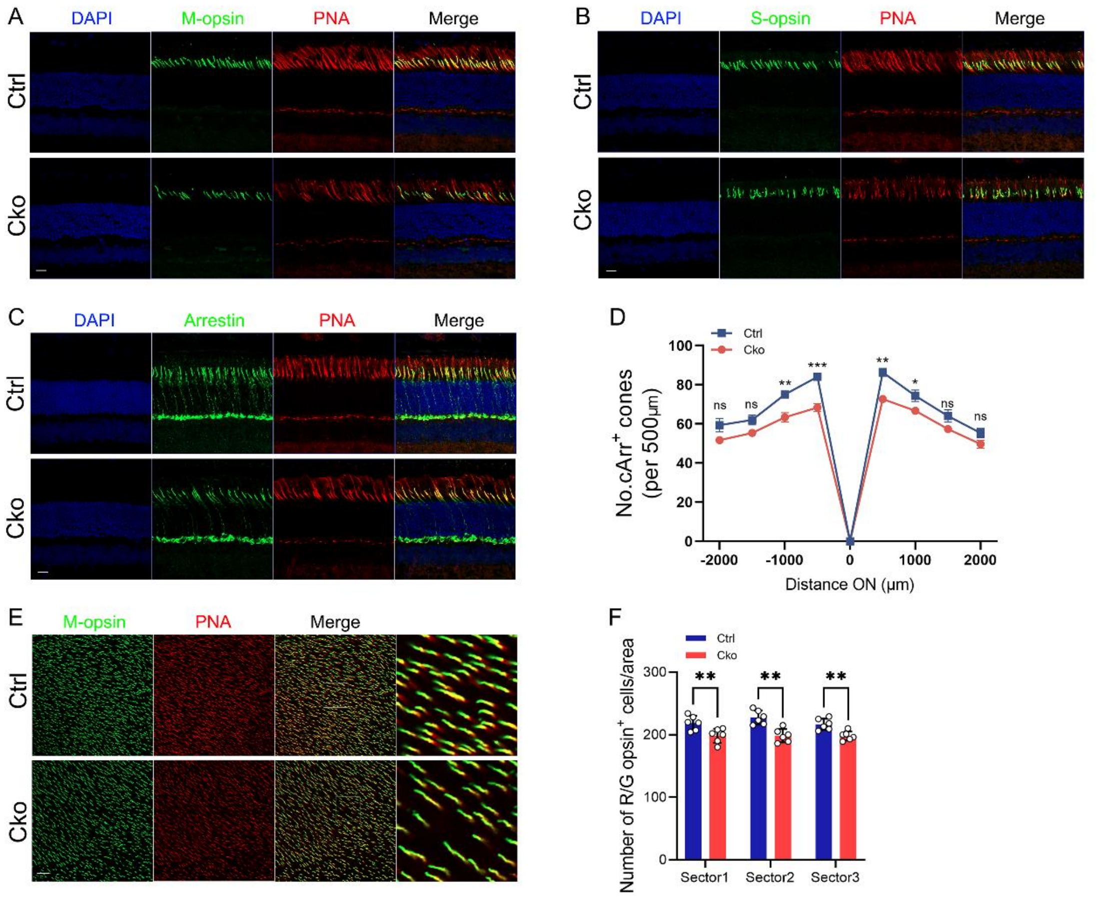

Figure S6. Early degeneration of cone photoreceptors in 3.5-month-old CKO mice. (A-C) Immunofluorescence staining of retinal cryosections from 3.5-month-old mice was performed to examine cone photoreceptors using antibodies against M-opsin (A), S-opsin (B), and Arrestin (C), with co-staining by the cone-specific marker PNA (red). Nuclei are stained with DAPI (blue). Scale bar, 25 µm. (D) Quantification of Arrestin-positive cone photoreceptors in both Ctrl and CKO retinas, with counts taken at 500 µm intervals across retinal sections. Statistical analysis revealed significant differences between groups (*: $P < 0.05$, **: $P < 0.01$, ***: $P < 0.001$, ns: $P > 0.05$). (E-F) Immunostaining of retinal whole-mounts from Ctrl and CKO mice using M-opsin (green) and PNA (red). Quantification analysis indicated a slight reduction in the number of cone photoreceptors in CKO retinas relative to Ctrl mice. Scale bar, 25 µm. Statistical significance is indicated by **: $P < 0.01$.

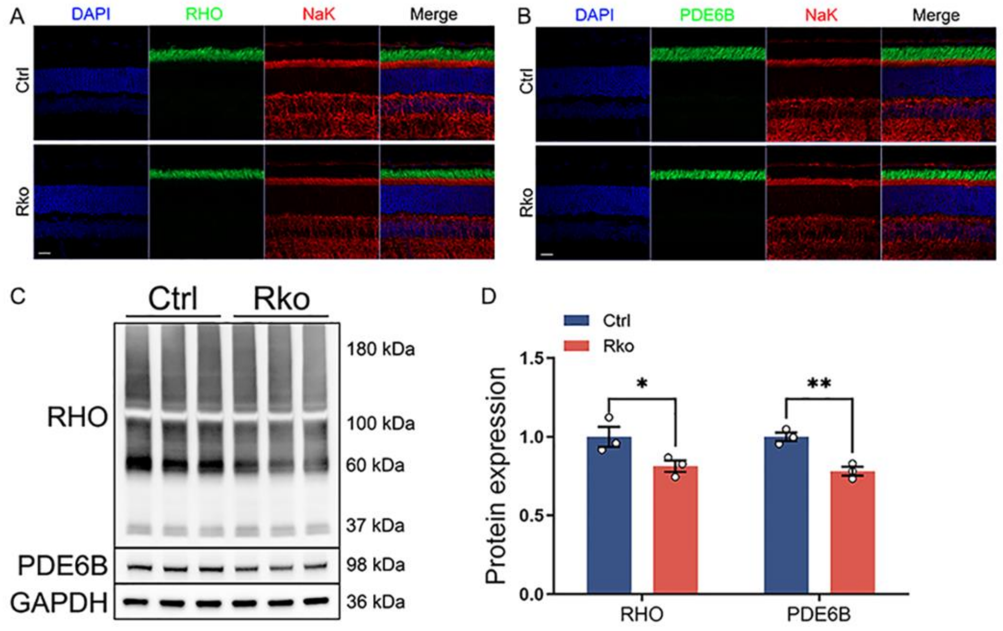

Figure S7. Early retinal degeneration in 2.5-month-old *Mettl3* RKO mice. (A, B) Retinal cryosections stained with DAPI for nuclei (blue). There was no significant difference in the thickness of the photoreceptor ONL or IS labeled with NaK ATPase antibody (red). However, the photoreceptor OS labeled with RHO (A) and PDE6B (B) (green) exhibit a noticeable thinning in RKO mice compared to that of Ctrl mice. Scale bar: 20 μm. (C, D) Western blot analysis of photoreceptor proteins (RHO and PDE6B) revealed a reduction in their expression levels in RKO mouse retinas, with RHO reduced by approximately 18% and PDE6B by approximately 22%, as indicated by grayscale value quantification. These data suggested that retinal degeneration has begun in RKO mice at 2.5 months of age. *: $p < 0.05$, **: $p < 0.01$

Table S1. The primer sequences used for RT-qPCR in this study.

Number	Primer	Sequence (5' to 3')
1	Rhodopsin-cDNA-F	AGTGAGGCTTCGGATTTTAACAG
2	Rhodopsin-cDNA-R	TGCTCAGTCCTTGAAAAGACAG
3	Prph2-cDNA-F	CCAACAACTCGGCGCACTA
4	Prph2-cDNA-R	CCTCCGGGTTAGACACACTCT
5	Pde6b-cDNA-F	GCAGCACTTTTTGAACTGGTG
6	Pde6b-cDNA-R	CATTGCGCTGGCGGTACATA
7	Gnat1-cDNA-F	GATGCCCGCACTGTGAAAC
8	Gnat1-cDNA-R	CCAGCGAATACCCGTCCTG
9	Guca1b-cDNA-F	GGAGGCGATTTACAAGCTGAA
10	Guca1b-cDNA-R	GCCGTCTCCATTCTCGTCC
11	Arl3-cDNA-F	ACCAGGAGGTGCGAATCCTA
12	Arl3-cDNA-R	ACCTTGTGATTGCACACTTTTGA
13	Unc119-cDNA-F	TTCGTCAGGTTCAAGATCCGT
14	Unc119-cDNA-R	CATGCGGAAGTTGTTGACCG
15	Rgs9-cDNA-F	TTCCCCGAGTCGCTTCATC
16	Rgs9-cDNA-R	CTGGGGTCTTGAGTGGTCT
17	Rgs9bp-cDNA-F	CAGGTGGGCGAGATGATTGAC
18	Rgs9bp -cDNA-R	CGCTCCTCTACCGAAATGC
19	Rs1-cDNA-F	ATGCCACACAAGATTGAAGGC
20	Rs1-cDNA-R	TAAGGAGGTAGCTCCAGCAGA

Table S2. Primary and secondary antibodies used in this study.

Antibody	Dilution Rate (WB)	Dilution Rate (IHC)	Species	Company	Cat. No.
Anti-METTL3	1:1000	1:200	Rabbit	Abcam	ab195352
Anti- METTL14	1:2000	1:200	Rabbit	Sigma-Aldrich	SAB5700855
Anti-WTAP	1:2000	-	Rabbit	Proteintech	60188-1-AP
Anti-Rhodopsin	1:2000	-	Rabbit	Cell Signaling Technology（CST）	14825
Anti-Rhodopsin	-	1:200	Rabbit	CST	27182
Anti-PRPH2	1:2000	1:200	Rabbit	Proteintech	18109-1-AP
Anti-RGS9	1:2000	1:200	Rabbit	Proteintech	17970-1-AP
Anti-GNAT1	1:2000	-	Rabbit	Proteintech	55167-1-AP
Anti-PDE6B	1:2000	1:200	Rabbit	Proteintech	22063-1-AP
Anti-GUCA1B	1:2000	-	Mouse	Proteintech	22298-1-AP
Anti-UNC119	1:2000	-	Mouse	Proteintech	13065-1-AP
Anti-RS1	1:2000	-	Mouse	Proteintech	24430-1-AP
Anti-ARL3	1:2000	-	Mouse	Proteintech	10961-1-AP
Anti- GFAP	1:2000	1:200	Rabbit	CST	80788s
Anti- GAPDH	1:5000	-	Mouse	Proteintech	60004-1-Ig
Anti- NaK ATPase	-	1:500	Mouse	Thermo	MA3-928
Anti-M Opsin	-	1:500	Rabbit	Sigma-Aldrich	AB5405
Anti-Arrestin	-	1:500	Rabbit	Sigma-Aldrich	AB15282

Reagent	Dilution Rate (WB)	Dilution Rate (IHC)	Species	Company	Cat. No.
Alexa Fluor™ 594 Conjugated PNA	-	1:500	-	Thermo	L32459
Anti-Mouse IgG 594	-	1:500	Goat	Thermo	A-11005
Anti-Rabbit IgG 488	-	1:500	Goat	Thermo	A-11008
HRP-Anti-Mouse	1:5000	-	Goat	Proteintech	SA00001-1
HRP-Anti-Rabbit	1:5000	-	Goat	Proteintech	SA00001-2
DAPI	-	1:500	-	Abcam	ab104139
